# Comprehensive analysis of skin growth-related hub genes and microenvironment characterization in a mouse expanded skin model

**DOI:** 10.3389/fimmu.2024.1306353

**Published:** 2024-12-05

**Authors:** Yinmin Wang, Wenxiao Qi, Shun Yu, Xianyu Zhou, Xiuxia Wang, Fei Liu, Rui Jin, Xusong Luo, Qiangliang Ma, Lin Lu, Jun Yang

**Affiliations:** ^1^ Department of Plastic and Reconstructive Surgery, Shanghai Ninth People’s Hospital, Shanghai Jiao Tong University, School of Medicine, Shanghai, China; ^2^ Department of Sports Medicine, Shanghai Sixth People’s Hospital, Shanghai Jiao Tong University, School of Medicine, Shanghai, China; ^3^ Affiliated Hospital of Jiangnan University, Wuxi, Jiangsu, China; ^4^ The First People’s Hospital of the Lancang Lahu Autonomous County, Puer, Yunnan, China; ^5^ Department of Dermatology, Traditional Chinese Medicine Hospital, Ili Kazakh Autonomous State, Yining, China

**Keywords:** skin regeneration, tissue expansion, hub genes, microenvironment, mast cell (MC)

## Abstract

**Background:**

Mechanical stretch-mediated tissue expansion is effective for obtaining extra skin and soft tissue required for the repair of defects or reconstruction of surface organs. Understanding the cellular and molecular mechanisms and identifying hub genes and key cell types associated with skin expansion could help predict the success of skin growth during expansion procedures.

**Methods:**

We analyzed murine chip sequencing data and single-cell sequencing data available from the Gene Expression Omnibus database. Based on the differentially expressed and epithelial–mesenchymal transition-related genes, random forest and protein-protein interaction network analysis identified hub genes for predicting skin regeneration in tissue expansion. The fate of the cell subpopulations, expression of hub genes in different cell types, and their communication were also assessed.

**Results:**

Five genes, integrin beta 5 (*Itgb5*), tropomyosin 1 (*Tpm1*), secreted frizzled-related protein-1 (*Sfrp1*), *Notch1*, and insulin-like growth factor binding protein 2 (*Igfbp2*), were identified as having the greatest impact on prediction accuracy. These hub genes were primarily enriched in the Notch and phosphoinositide 3-kinase-AKT pathways. Immune cell infiltration analysis further revealed that mast cell infiltration was significantly higher in the expanded skin group than that in the control group. According to single-cell data, the interactions between epithelial cells, stem cells, and other cell types were higher in the expanded skin group than those in the control group. Moreover, *Tpm1, Sfrp1*, and *Notch1* were highly expressed in all epithelial and stem cell subgroups.

**Conclusions:**

The hub genes, *Notch1, Tpm1 and Sfrp1*, and their associated signaling pathways such as Notch and Wnt signaling and functions in key cell subsets highlight prospective therapeutic strategies to enhance skin growth under mechanical expansion. Moreover, mast cell activation and infiltration may trigger immune responses in the expanded skin, which requires further investigation.

## 1 Introduction

Mechanical stretch-mediated tissue expansion is an effective procedure to obtain extra skin and soft tissue via skin regeneration to aid in repairing defects or reconstruction of surface organs in plastic and reconstructive surgeries (1, 2). During this process, an expander is inserted underneath the skin and soft tissue. However, complications, such as expander exposure, may occur if skin regeneration is insufficient (3). Thus, understanding the cellular and molecular mechanisms and identifying hub genes and key cell types associated with skin expansion will help evaluate the degree of skin growth during tissue expansion and provide insights into insufficient skin expansion. Furthermore, the analysis of single-cell and transcriptome data may elucidate the mechanisms employed by different skin cell populations in response to mechanical stretching.

Epithelial–mesenchymal transition (EMT) is a key regulator of skin regeneration during skin expansion as shown in previous study (4). EMT is a cellular process vital for wound healing and embryogenesis, during which, the cell–extracellular matrix and cell–cell interactions become remodeled, leading to a new transcriptional program that promotes the mesenchymal fate of epithelial cells. Thus, EMT related genes were chosen in this study for further analysis. Furthermore, EMT may impact the infiltration and activation of various immune cell types in the stroma. Indeed, the relationship between immune response and EMT has been reported in studies on wound healing (5, 6). In particular, cytokines, such as interleukin (IL)-6, tumor necrosis factor-alpha (TNF-α), and transforming growth factor-beta (TGF-β), are released by activated effector T cells or macrophages and facilitate EMT (7–9). Meanwhile, immune cells in the expanded skin microenvironment influence skin regeneration. That is, the polarization of macrophages from the pro-inflammatory M1 phenotype to the immunosuppressive M2 response is necessary for skin regeneration during tissue expansion (10). However, following mechanical stretching, high M1 polarization-related gene expression is observed *in vitro* (11). Hence, considering the prominent role played by monocytes/macrophages in tissue repair and regeneration, it is necessary to investigate their roles in skin regeneration during tissue expansion and characterize their communication with resident cells (12). Moreover, immune infiltration analysis in expanded skin may provide insights into the mechanism underlying the interactions between immune responses and skin regeneration during tissue expansion.

Previous studies have illustrated the mechanisms of skin regeneration and renewal during tissue expansion, *in vivo* and *in vitro* (4, 13). Based on these studies, herein, we comprehensively analyzed chip and single-cell RNA sequencing (scRNA-seq) data from the Gene Expression Omnibus (GEO) database. Based on differentially expressed genes (DEGs) and EMT-related genes, a random forest model and protein–protein interaction (PPI) network analysis was performed to identify a group of hub genes capable of predicting skin regeneration success during tissue expansion. Furthermore, the fate of cell subpopulations, expression of hub genes in different cell types, and their communication were evaluated.

The findings of this study will help evaluate the degree of skin growth during tissue expansion and provide insights into insufficient skin expansion.

## 2 Materials and methods

### 2.1 Data acquisition and processing

The scRNA-seq dataset GSE146637 was downloaded from the official GEO database (https://www.ncbi.nlm.nih.gov/geo/) for further analysis (14). The species was Mus musculus, and an Illumina HiSeq 4000 detection platform was used. Samples were obtained from the skin tissues of mice in the two expanded skin groups and one control group. The raw data included 12,309 cells. Quality control of single-cell data was performed, and the processing standards were identified according to the following criteria: (1) gene was expressed in at least 10 cells; (2) at least 200 genes per cell; (3) total number of counts in each cell was between 0 and 30,000; (4) the number of genes expressed per cell was between 1,200 and 5,000; (5) mitochondrial gene content was < 10%; and (6) ribosomal gene content was between 10% and 50%. The final expression matrix contained 12,263 cells and 8,958 genes, with 7,608 and 4,655 cells in the expanded and control groups, respectively.

Chip data were obtained from the GEO website. Among them, the GSE126231 dataset was from the Affymetrix HT MG-430 PM Array Plate platform, including the chip expression matrix and sample information data of eight Mus musculus samples (13). The expression matrix and sample information data of the three expanded skin groups and the three control samples were selected for analysis. GSE186773 was obtained from the Affymetrix Clariom S Assay, Mouse (Includes Pico Assay) platform (species: Mus musculus) (15) comprising 10 samples. The microarray sequencing expression matrix and sample information data of four samples each from the untreated expanded skin group and four control groups were evaluated. Each dataset contained an expression matrix and corresponding sample information data (see **Supplementary Table 1**).

### 2.2 Identification of EMT-related genes

We searched the Ensembl database (https://asia.ensembl.org/index.html) with the keyword “EMT” and obtained 251 EMT-related genes (16). In addition, we collected multiple EMT gene sets from the Mouse Genome Informatics (MGI) database (https://www.informatics.jax.org/) and Molecular Signatures Database (MsigDB) (17). After removing duplicates, all genes were included in the subsequent analyses, and 362 EMT-related genes were identified (see **Supplementary Table 2**).

### 2.3 Integration of datasets after batch effect removal

To expand the sample size and ensure the reliability of the experimental results, we used the ComBat function in surrogate variable analysis (SVA; version 3.42.0) to remove the batch effects of GSE126231 and GSE186773 Chip datasets; the co-expressed genes were retained (18). The resulting integrated expression matrix was used for subsequent analyses.

### 2.4 Variance analysis

Linear Models for Microarray Data (limma) (version 1.34.0) was used to perform differential analysis on the expression matrix of all microarray data to identify DEGs between the expanded skin and control groups (19). The parameters for DEGs included a log fold change [log(FC)] |logFC|> 0.5 and *P*-value < 0.05. The DEGs and EMT-related genes were then intersected to obtain EMT-related DEGs (EMTRDEGs) for further study.

### 2.5 Gene Ontology (GO) and Kyoto Encyclopedia of Genes and Genomes (KEGG) analysis

GO analysis is a common method for conducting large-scale functional enrichment studies on biological processes (BPs), molecular functions (MFs), and cellular components (CCs) (20). KEGG is a widely used database that stores information on genomes, biological pathways, diseases, and drugs (21). The R package clusterProfiler (version 4.2.2) was used to perform GO and KEGG annotation analyses of EMTRDEG in the integrated dataset (22). The entry screening criterion was *P* < 0.05, a false discovery rate value (q-value) < 0.05 was considered statistically significant, and the Benjamini-Hochberg P-correction method was used.

### 2.6 Chromosome location information

We used RCircos (version 1.2.2) to determine the locations of EMTRDEGs in mouse chromosomes (23). Using the on-chromosome gene mapping information built into the University of California in Santa-Clara UCSC. Mouse. GRCm38. CytoBandIdeogram data, we selected and visualized the EMTRDEGs.

### 2.7 Establishment of diagnostic model for skin growth

We used the previously obtained EMTRDEGs as candidate genes to construct diagnostic models. The randomForest function in randomForest (version 4.7) was used to perform random forest variable screening based on the integrated dataset to construct diagnostic models. According to the random forest machine learning algorithm, the EMTRDEGs were weighted and scored, and the top five genes with the highest interpretation degree for the prediction model were selected as predictors. Only the diagnostic markers were retained, and the model was reconstructed. Subsequently, we used the receiver operating characteristic (ROC) curve function in package pROC (version 1.18.0) to draw the ROC curve of the diagnostic model (24). The nomogram function in root mean square (version 6.3-0) was employed to draw the nomogram, and the model was calibrated with a calibration function to test the model prediction accuracy.

### 2.8 Weighted correlation network analysis (WGCNA)

WGCNA (version 1.71) was performed on all genes in the integrated dataset (25). The constant-height tree cut cutreeStatic function was used to filter out outlier samples. We defined a cut height of 140 mm as an outlier sample with a large deviation for elimination. The pickSoftThreshold function selected the first parameter R2 > 0.85 as the soft threshold for the subsequent construction of gene modules. Next, we clustered the co-expressed genes using the blockwiseModules function to obtain gene modules. The important parameters were set as follows: (1) maximum block size = 16,000; (2) minimum module size = 200; (3) minimum CoreKME size = 200/3; (4) merge cut height = 0.15; (5) deep split = 4. The default values were used for the remaining parameters.

Spearman correlation analysis was performed between the final gene modules and their characteristic values and the grouping status of each sample (expanded skin group =“1,” normal control group =“0”). The most relevant gene modules significantly associated with skin growth in the expanded group were screened for *P* < 0.05.

### 2.9 PPI network construction and identification of hub genes

The previously obtained skin growth-related gene modules were intersected with diagnostic markers to obtain skin growth-related diagnostic markers (SGRDMs). We then constructed a PPI network using the Search Tool for the Retrieval of Interacting Genes/Proteins (STRING) (https://cn.string-db.org/) database (26), selected the top 20 genes with the highest network connectivity, excluded the genes that did not exist in the expression matrix of the integrated dataset, and formed hub genes together with SGRDMs. Cytoscape software (version 3.9.1) was used to visualize gene networks (27).

Subsequently, we used Network Analyst (https://www.networkanalyst.ca/) to construct a micro-RNA (miRNA) and transcription factor (TF) interaction network of hub genes (28). Finally, network connectivity was calculated and visualized using the CytoHubba plugin.

### 2.10 Immune cell infiltration

The immune cell composition (ImmuCC) model (http://218.4.234.74:3200/immune/) is based on the principle of linear support vector regression to deconvolve the transcriptome expression matrix or chip expression matrix to estimate the composition and abundance of immune cells in various tissues (29). We uploaded the integrated dataset of this study to ImmuCC for evaluation and analyzed the infiltration fraction of 25 immune cells in different samples and the difference between expanded skin and control groups. Corrplot (version 0.92) was used to map correlations between the 25 immune cells and hub genes. We then screened for statistically significant correlations between immune infiltration scores and hub gene expression and plotted the correlations using the scatterplot function. Statistical significance was set at *P* < 0.05.

### 2.11 Dimensionality reduction, clustering, and grouping of single-cell data

We analyzed the single-cell data using Seurat (version 4.0.5) (30). Next, the sequencing depth of the GSE146637 dataset was normalized with the “NormalizeData” function, which is the default LogNormalize, and detected 1,500 variable features of the dataset using the variance-stabilizing transformation (VST) method by calling the FindVariableFeatures function. We then scaled the data using ScaleData to exclude the effects of sequencing depth. Subsequently, we selected 12 significant principal components (PCs) for dimensionality reduction and clustering using the FindClusters function (resolution = 0.2). The final visualization was performed using a Uniform Manifold Approximation and Projection (UMAP). The cells were then divided into six clusters.

### 2.12 Identification of characteristic genes and cell type annotation

We used the FindAllMarkers function in Seurat to identify characteristic genes specifically expressed in each cell population. A gene satisfying |logFC|> 0.25 and adjusted *P* < 0. 05 was considered a characteristic gene. The characteristic genes of each cell group were ranked according to |logFC|, and the genes with the highest ranking were selected to characterize and annotate each group. Each cell population was type-annotated by examining the expression of reported marker genes in the population.

### 2.13 Cell subpopulation identification

Stem cells, endothelial cells, epithelial cells, and fibroblasts were extracted to identify the cell subpopulations. Further analyses of the four cell populations were performed using the Seurat protocol. The important parameters were set as follows: Stem cells and Endothelial cells: The FindVariableFeatures function used the VST method to detect 1,200 variable features of the dataset, PCs = 8, resolution = 0.1; Epithelial cells: The FindVariableFeatures function used the VST method to detect 800 variable features of the dataset, PCs = 6, resolution = 0.1.

### 2.14 Cell communication

CellChat (version 1.1.3) extrapolates the strength of interactions between different cells based on single-cell expression matrices and ligand-receptor pair information recorded in the in-house CellChat mouse database (31). We performed cell communication analysis on all the cells and visualized them.

### 2.15 Gene set variation analysis (GSVA)

We downloaded mouse HALLMARK gene sets (32) for GSVA from MSigDB (https://www.gsea-msigdb.org/gsea/msigdb/). The HALLMARK gene sets comprised 50 important gene sets related to biological functions. We selected HALLMARK gene sets based on the GSE146637 dataset, calculated the biological activity of each cell population, and identified gene sets with differences in functional activity between the expanded skin groups and controls.

### 2.16 Cell differentiation trajectory inference

Monocle (version 2.22.0) was used to construct quasi-temporal trajectories for four cell populations: stem cells, endothelial cells, epithelial cells, and fibroblasts (33). The genes used for trajectory inference were characteristic of all four cell groups.

### 2.17 TF activity assay

The discriminant regulon expression analysis DoRothEA resource (version 1.6.0) is a method for inferring the activity of transcription factors in each cell based on an expression matrix (34). We calculated the activity of TFs in stem cells, endothelial cells, epithelial cells, and fibroblast cell groups and identified cell subgroup-specific TFs.

### 2.18 Statistical analysis

All data processing and analyses were performed using the R software (version 4.1.1). To compare two groups of continuous variables, statistical significance was estimated using the independent Student’s *t*-test for normally distributed variables. Differences between non-normally distributed variables were analyzed using the Mann–Whitney *U*-test (i.e., Wilcoxon rank-sum test). The chi-squared test or Fisher’s exact test was used to compare and analyze the statistical significance of the difference between two groups of variables. All statistical *P* values were two-sided, and a *P* < 0.05 was considered significant.

## 3 Results

### 3.1 Flow chart of the experimental protocol

**Figure 1:**
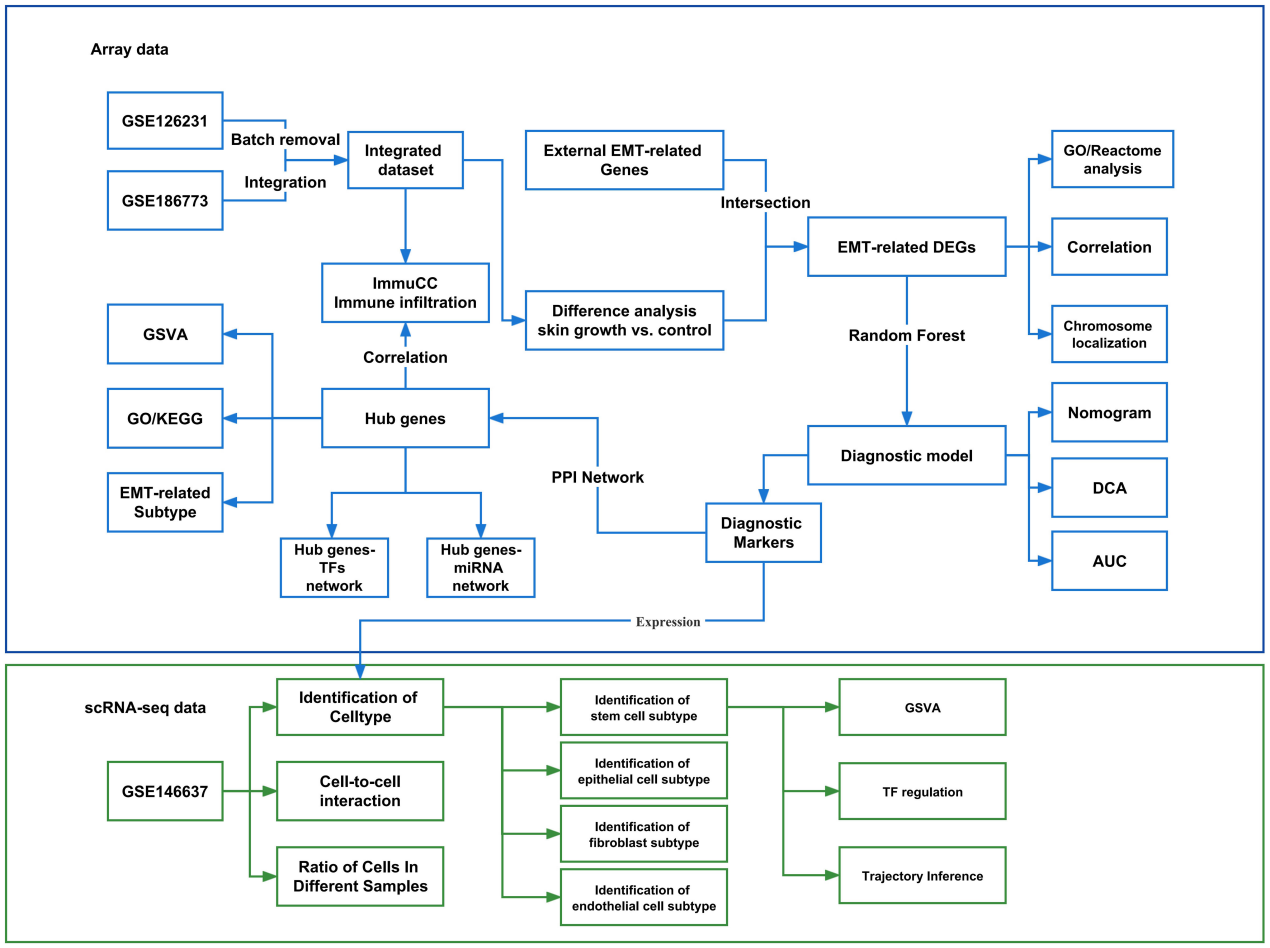
Flow chart of the experimental protocol. GSVA, Gene set variation analysis; GO, Gene Ontology; KEGG, Kyoto Encyclopedia of Genes and Genomes; EMT, Epithelial-mesenchymal transition; PPI, Protein-protein interaction network; TF, Transcription factors. DEGs, Differentially expressed genes; DCA, Decision Curve Analysis; AUC, Area under curve.

The flow chart of this study is shown in **Figure 1**.

### 3.2 Chip data integration

To expand the sample size, include more analytical data, and have a more comprehensive characterization of skin growth, we integrated the Chip datasets GSE126231 and GSE186773 via de-batching. Before the process, the total expression of the samples in the two datasets exhibited differences, as reflected in **Supplementary Data Sheet 2**. Samples from the different datasets were divided into two populations (**Supplementary Data Sheet 2**). Subsequently, datasets GSE126231 and GSE186773 were processed to remove the batch effect using the R package sva function to obtain combined datasets. The datasets before and after batch effect removal were compared using a distribution box plot (**Supplementary Data Sheet 2**) and PC analysis (PCA) plots (**Supplementary Data Sheet 2**). Fourteen samples were included in the integrated dataset, including seven from the expanded skin group and seven from the control group.

### 3.3 Identification of EMT-related DEGs

**Figure 2:**
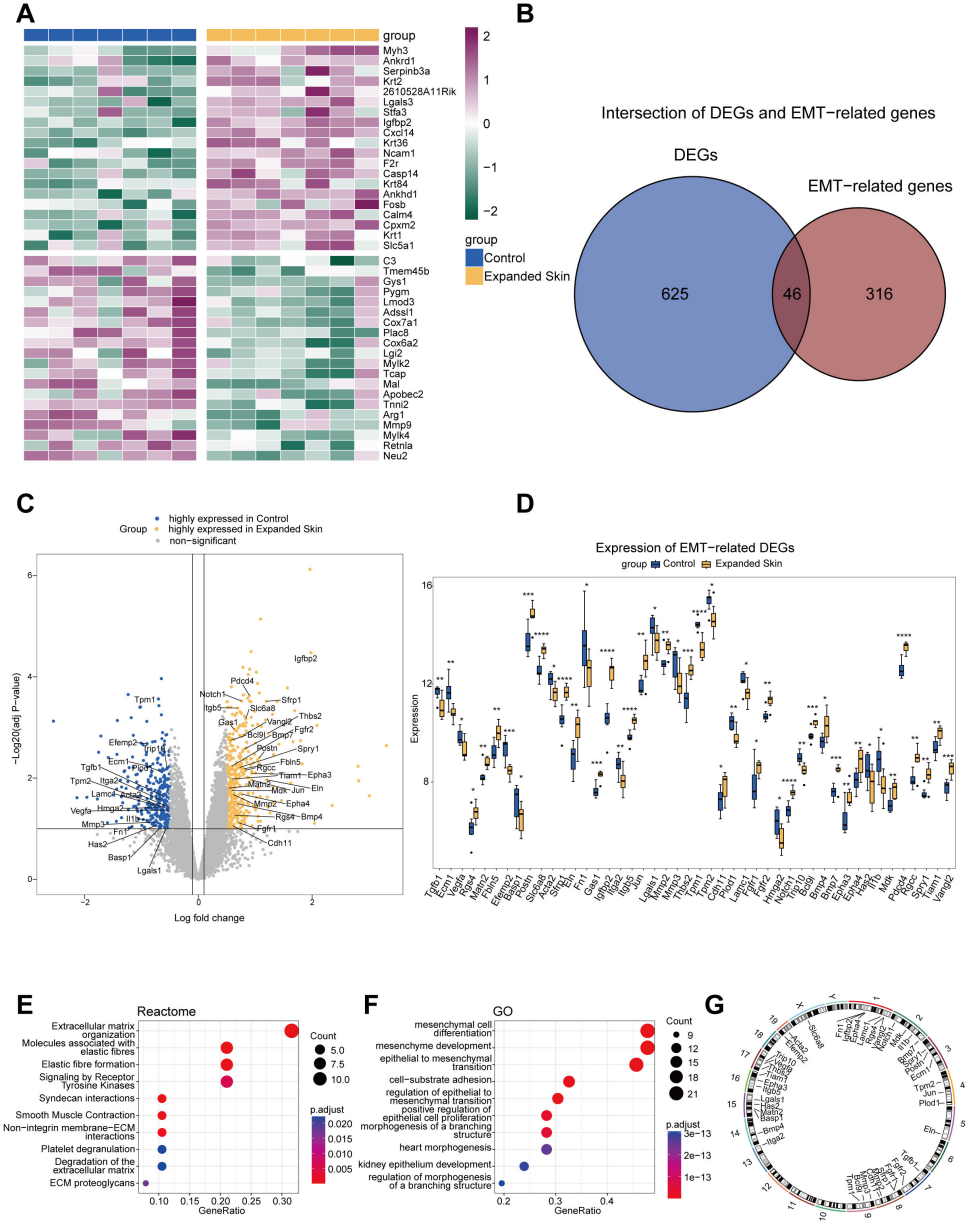
Identification of EMTRDEGs based on the integrated dataset. **(A)** DEGs of logFC top 20 were upregulated in expanded and control groups, respectively. Purple indicates high expression, while green indicates low expression. **(B)** The intersection of 671 DEGs and 362 EMT-related genes yielded 46 EMTRDEGs. **(C)** The volcano plots show the expression of 46 EMTRDEGs in two groups. **(D)** Box plots show the differences in the expression of 46 EMTRDEGs between two groups. *P < 0.05, **P < 0.01, ***P < 0.001, ****P < 0.0001. **(E)** Reactome pathway enrichment map. The size of the black circle indicates the number of EMTRDEGs to which an entry is enriched. **(F)** GO pathway enrichment map. The size of the black circle indicates the number of EMTRDEGs to which an entry is enriched. **(G)** Location information of EMTRDEGs on different human chromosomes. EMTRDEGs, EMT-related DEGs.

To investigate the differences between the expanded and control groups, we identified DEGs based on the integrated dataset. Among them, 379 DEGs were significantly upregulated in the expanded group and 292 DEGs were significantly downregulated (see **Supplementary Data Sheet 3**). We then selected 20 DEGs with the top ∣logFC∣in expanded and control group, respectively, to construct a heat map and found significant differences in gene expression between the expanded and control groups (**Figure 2A**).

To further evaluate the relationship between DEGs and EMT-related genes, we intersected the DEGs with EMT-related genes obtained from the Ensemble, MGI, and MsigDB (**Figure 2B**). A total of 46 EMTRDEGs were obtained (*P* < 0.05, **Figure 2C**). Among these, 28 were upregulated in the expansion group and 18 were upregulated in the control group (*P* < 0.05, **Figure 2D**). To further assess the biological function of EMTRDEGs and their roles in signaling pathways, we performed GO and Reactome enrichment analyses on all 46 EMTRDEGs. The results showed significantly enriched ECM organization and elastic fiber formation pathways (*P* < 0.05; **Figure 2E** and **Supplementary Table 4**). GO results further revealed that the EMTRDEGs were significantly enriched in mesenchymal cell differentiation pathways related to cell differentiation other than EMT pathways (*P* < 0.05; **Figure 2F** and **Supplementary Table 4**). In addition, chromosome mapping results showed that the EMTRDEGs were located on multiple chromosomes, suggesting that they might have different biological functions (**Figure 2G**).

### 3.4 Correlation analysis of EMTRDEGs

**Figure 3:**
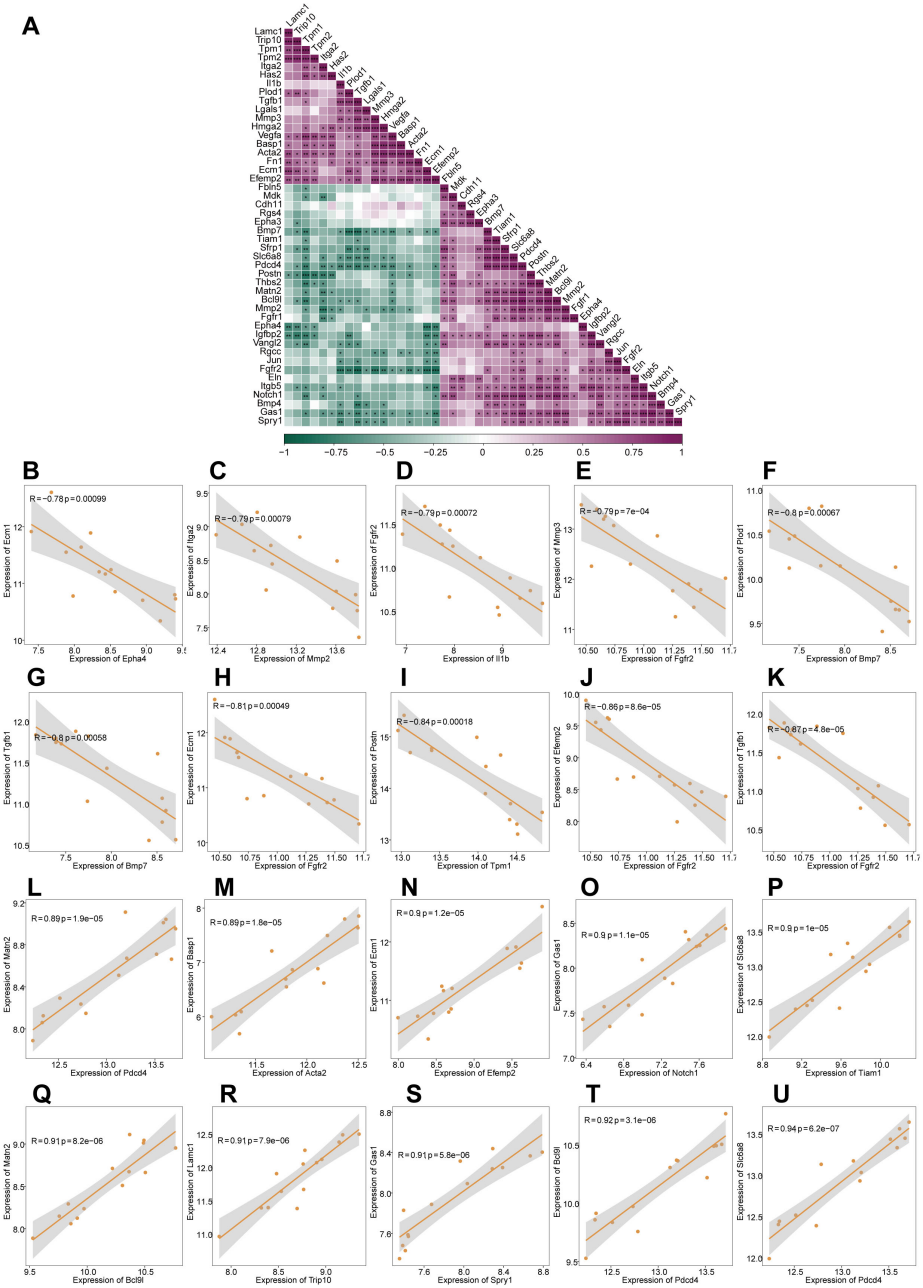
Correlation analysis of EMTRDEGs based on the integrated dataset. **(A)** Correlation heat map of 46 EMTRDEGs. Purple represents a positive correlation and green represents a negative correlation. *P < 0.05, **P < 0.01, ***P < 0.01. **(B–U)** Scatter plot of EMTRDEG correlations (|r| >0.7 and *P* < 0.05).

We performed gene expression correlation analysis on the 46 EMTRDEGs and found that the expression levels of most EMTRDEGs were significantly positively correlated (*P* < 0.05, *r* > 0), whereas those of others were significantly negatively correlated (*P* < 0.05, *r* < 0; **Figure 3A**). In addition, the expression of various EMTRDEG pairs, namely, *Epha4* and *Ecm1* (**Figure 3B**), *Mmp2* and *Itga2* (**Figure 3C**), *Il1b* and *Fgfr2* (**Figure 3D**), *Fgfr2* and *Mmp3* (**Figure 3E**), *Bmp7* and *Plod1* (**Figure 3F**), *Bmp7* and *Tgfb1* (**Figure 3G**), *Fgfr2* and *Ecm1* (**Figure 3H**), *Tpm1* and *Postn* (**Figure 3I**), *Fgfr2* and *Efemp2 (* **Figure 3J**), and *Fgfr2* and *Tgfb1* (**Figure 3K**) were negatively correlated (*P* < 0.001, *r* < -0.7). The expression levels of *Pdcd4* and *Matn2* (**Figure 3L**), *Acta2* and *Basp1* (**Figure 3M**), *EfemP2* and *Ecm1* (**Figure 3N**), *Notch1* and *Gas1* (**Figure 3O**), *Tiam1* and *Slc6a8* (**Figure 3P**), *Bcl9l* and *Matn2* (**Figure 3Q**), *Trip10* and *Lamc1* (**Figure 3R**), *Spry1* and *Gas1* (**Figure 3S**), *Pdcd4* and *Bcl9l* (**Figure 3T**), and *Pdcd4* and *Slc6a8* (**Figure 3U**) were positively correlated (r > 0.8, p < 0.001). This high degree of correlation between EMTRDEG expression patterns suggests that they may function concertedly to influence skin growth.

### 3.5 Construction of skin growth diagnosis model in skin-expansion tissue based on EMTRDEGs

**Figure 4:**
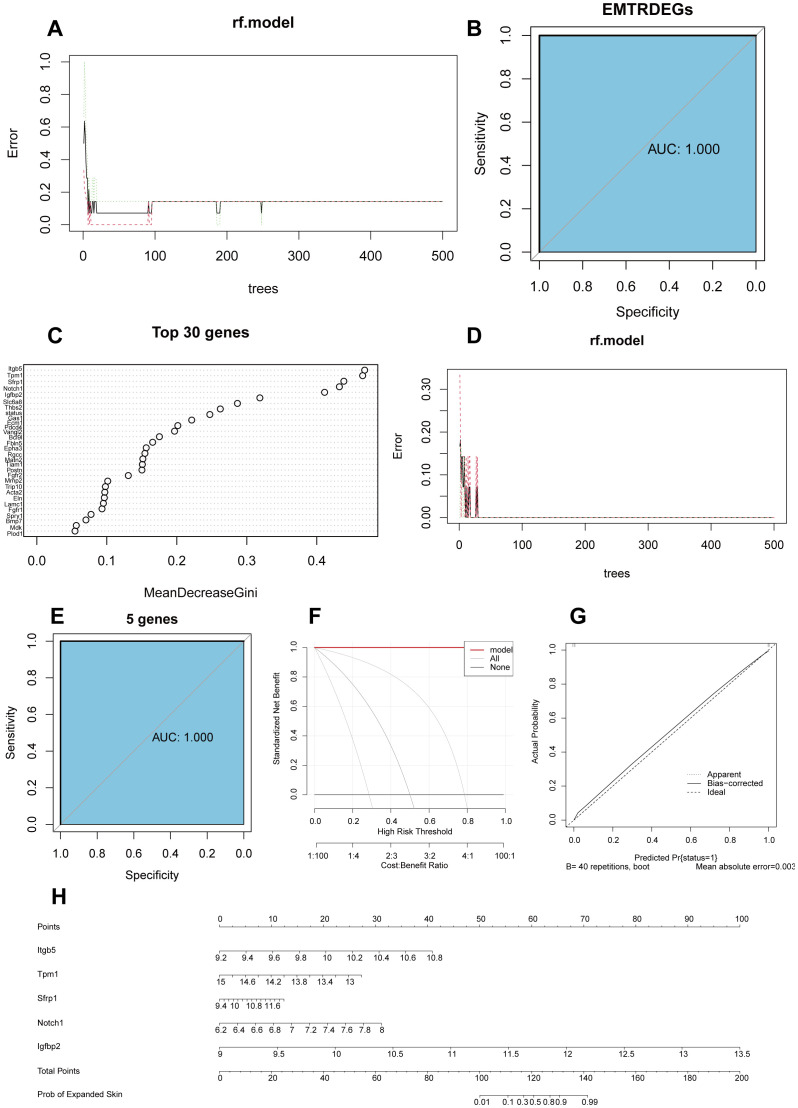
Construction of a diagnostic model based on the integrated dataset and EMTRDEGs. **(A)**. Error curves of 46 EMTRDEGs in random forest classification model. **(B)** ROC curves based on diagnostic models for all EMTRDEGs. The area under the curve (AUC) represents the accuracy of the model. **(C)** Weight of the first 30 EMTRDEGs in the random forest classification model. The larger the MeanDecreaseGini, the more important the variable. **(D)** Error curves of five diagnostic markers in random forest model. **(E)** ROC curve of diagnostic model based on diagnostic markers. **(F)** DCA plot of the diagnostic model. The ordinate is the net profit, and the abscissa is the threshold probability. **(G)** Calibration curve of random forest model based on integrated data set. **(H)** Nomogram plot based on integrated dataset. ROC, receiver operating characteristic; DCA, decision curve analysis.

To determine the diagnostic value of the 46 EMTRDEGs in the integrated dataset, we constructed a diagnostic model based on the integrated dataset using random forest analysis. We found that when the n-tree parameter was > 300, the model error rate tended to be stable (**Figure 4A**). In addition, the accuracy of skin growth prediction based on the 46 EMTRDEGs was 100% (area under curve [AUC] = 1) (**Figure 4B**).

The MeanDecreaseGini in random forests calculates the effect of each variable on the heterogeneity of the observations at each node of the classification tree. Comparing the importance of the variables, the larger the value, the greater the importance of the variable. We found that the top five genes (integrin subunit beta 5 (*Itgb5*), tropomyosin 1 (*Tpm1*), secreted frizzled related protein 1 (*Sfrp1*)*, Notch1*, and insulin-like growth factor binding protein 2 (*Igfbp2*)) were of considerable importance for predicting skin growth (**Figure 4C**). Accordingly, these five genes were designated diagnostic markers and included in the random model for diagnostic prediction. The resulting random forest classification model maintained a constant error rate when n-tree parameter was > 100 (**Figure 4D**). The predictive accuracy of the five diagnostic markers for skin growth was 100% (AUC = 1) (**Figure 4E**). Subsequently, a calibration curve was drawn using decision curve analysis (DCA; **Figure 4F**) and calibration analysis (**Figure 4G**). The model prediction based on the results was evaluated based on the conformity between the actual probability and the probability predicted by the model under different circumstances. The results showed that the prediction efficiency of the model was high, and the prediction results were highly consistent with the actual skin growth of the model. Finally, we plotted nomograms for the five genes whose expression levels were good predictors of skin growth probability (**Figure 4H**).

### 3.6 Identification of gene modules associated with skin growth using WGCNA

**Figure 5:**
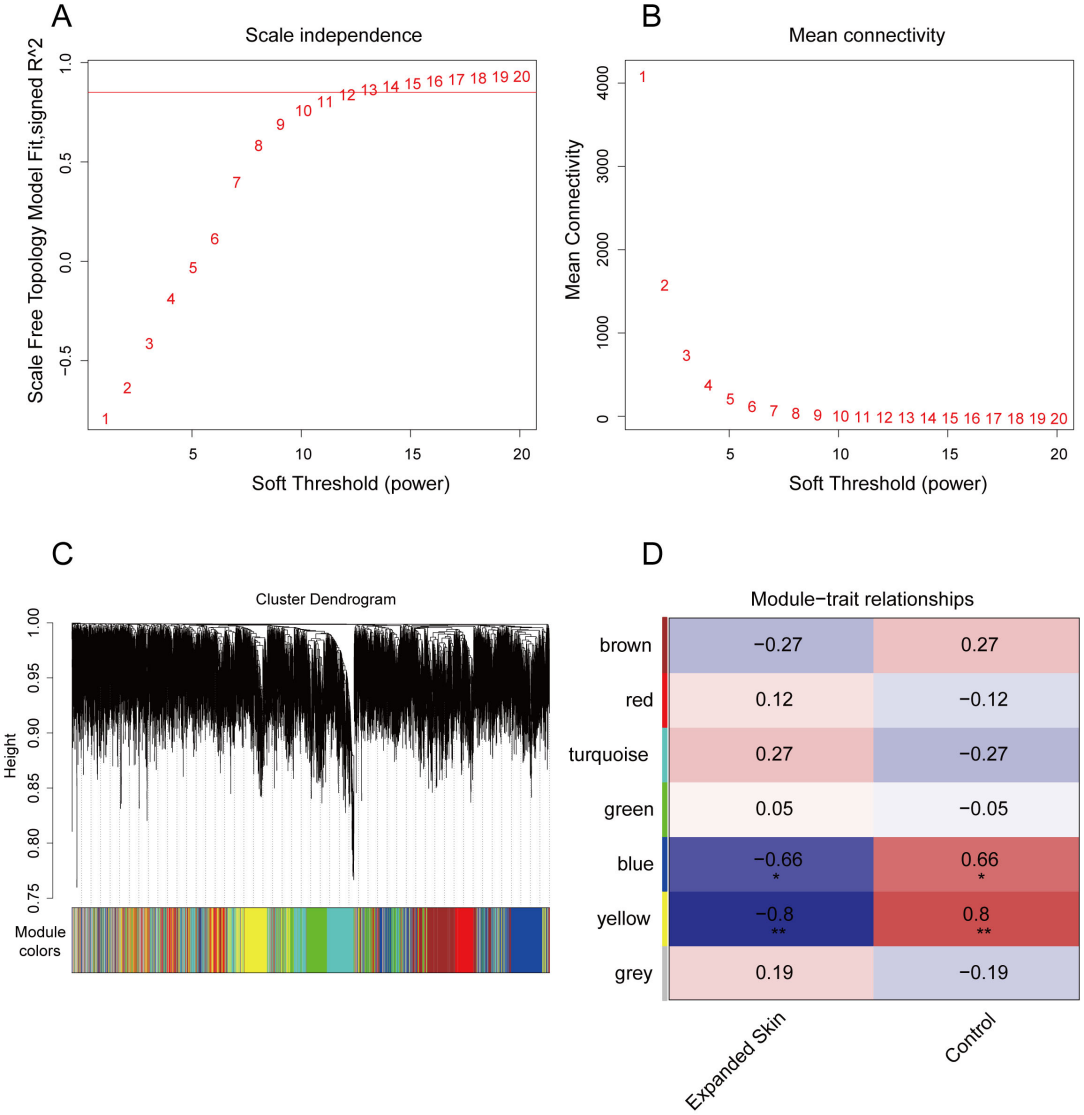
WGCNA screening of skin growth-related gene modules based on integrated datasets. **(A)** Optimal soft threshold determined as shown with the scale-free fit index as a function of the soft-thresholding power. **(B)** Average connectivity of undirected networks corresponding to different transition thresholds. **(C)** Formation of different gene modules. **(D)** Heatmap of Spearman's correlations of seven gene modules with expanded skin and control groupings. Red indicates positive correlation, while blue indicates negative correlation. *P < 0.05, **P < 0.01. WGCNA, weighted gene co-expression network analysis.

In addition to the diagnostic markers, we used WGCNA to identify modules that were highly correlated with skin growth for subsequent analysis. To this end, we used the expression matrix of the integrated dataset of all genes as the input file and set the optimal soft threshold to 12 (**Figure 5A**). A scale-free network (**Figure 5B**) was constructed, and the topological matrix was calculated. Hierarchical clustering was performed (**Figure 5C**). Seven gene modules were obtained by setting the minimum number of modular genes to 200 to construct gene modules (**Figure 5D**). We further correlated these modules with the expanded and control groups and found that the blue and yellow modules were significantly negatively correlated with the expanded growth group (*P* < 0.05, *r* = -0.66 and -0.8, respectively) and significantly positively correlated with the control group (*P* < 0.05, *r* = 0.66 and 0.8, respectively). Therefore, we identified the blue and yellow modules as core modules (**Figure 5D**, **Supplementary Table 5**).

### 3.7 Identification of hub genes

To further investigate the relationship between diagnostic markers and skin growth, we intersected the five diagnostic markers with the core modules and found the five diagnostic markers were associated to core modules related to skin growth processes. (**Supplementary Data Sheet 2**).

Next, we constructed a PPI network of these five genes using the STRING database to identify the hub genes associated with skin growth. We selected the top 20 genes with the highest network connectivity (excluding genes not included in the expression matrix of the integrated dataset) by incorporating the five diagnostic markers to form 23 hub genes, including catenin beta 1 (*Ctnnb1*), *Notch1*, insulin-like growth factor 1 (*Igf1*), nicastrin (*Ncstn*), presenilin 1 (*Psen1*), recombination signal binding protein for immunoglobulin kappa J region (*Rbpj*), *Psen2, Igf1* receptor (*Igf1r*), *Igf2*, amyloid beta precursor protein (*App*), integrin subunit beta 3 (*Itgb3*), integrin subunit alpha V *(Itgav*), *Numb*, hes related family bHLH transcription factor with YRPW motif 1 (*Hey1*)*, Igfbp4, Itgb5, Igfbp1, Igfbp3, Igfbp2*, mastermind like transcriptional coactivator 1 (*Maml1*), troponin T2 (*Tnnt2*), *Tpm1*, and *Sfrp1* (**Supplementary Data Sheet 2**). Among them, *Notch1, Ncstn, Ctnnb1*, and *Psen1* showed high connectivity, whereas *Igfbp3, Sfrp1, Tnnt2*, and *Tpm1* exhibited low connectivity (**Supplementary Data Sheet 2**).

### 3.8 Construction of the interaction hub gene network with miRNAs and TFs

To further evaluate the interactions between hub genes and miRNAs or TFs, we constructed an miRNA and TF interaction network with hub genes using the NetworkAnalyst database. We found that hub genes interacted with multiple miRNAs, among which *Itgav, Igf1*, and *Igf1r* were the most connected, suggesting that they might have the strongest interactions with miRNAs (**Supplementary Data Sheet 2**). In the TF interaction network, *Itgb3, Notch1*, and *Rbpj* were the most connected (**Supplementary Data Sheet 2**).

### 3.9 Hub gene enrichment analysis

Next, we performed GSVA of the hub genes to explore the differences in gene set activity between the expanded and control groups. The results showed no significant differences in the activity of the gene set composed of hub genes between the two groups (*P* = 0.21; **Supplementary Data Sheets 2**, **4**). The KEGG results showed that hub genes were mainly enriched in the Notch, PI3K-AKT, and thyroid hormone signaling pathways, which are important pathways related to cell proliferation (**Supplementary Data Sheet 2**, **Supplementary Table 6**). In addition, the GO results showed that hub genes were mainly enriched in the insulin-like growth factor receptor signaling pathway, regulation of the insulin-like growth factor receptor signaling pathway, and muscle cell proliferation (**Supplementary Data Sheet 2**, **Supplementary Table 6**). KEGG analysis revealed the locations of hub genes in important pathways and their potential upstream and downstream regulatory relationships. We found that *Itgb3, Itgav*, and *Itgb5* were enriched in ECM-receptor interactions and that these genes might be involved in ECM remodeling (**Supplementary Data Sheet 2**). *Notch1, Ncstn, Psen1, Rbpj, Psen2, Numb, Hey1*, and *Maml1* were enriched in the Notch signaling pathway, and these genes were also involved in the mitogen-activated protein kinase signaling pathways (**Supplementary Data Sheet 2**). *Igf1, Igf1r, Igf2, Itgb3, Itgav*, and *Itgb5* were also enriched in the PI3K-Akt signaling pathway, indicating that they play an important role in cell proliferation (**Supplementary Data Sheet 2**). In addition, we found that *Ctnnb1, Notch1, Itgb3*, and *Itgav* were enriched in the thyroid hormone signaling pathway and that thyroid hormones also regulate cell growth (**Supplementary Data Sheet 2**).

### 3.10 Immune cell infiltration

**Figure 6:**
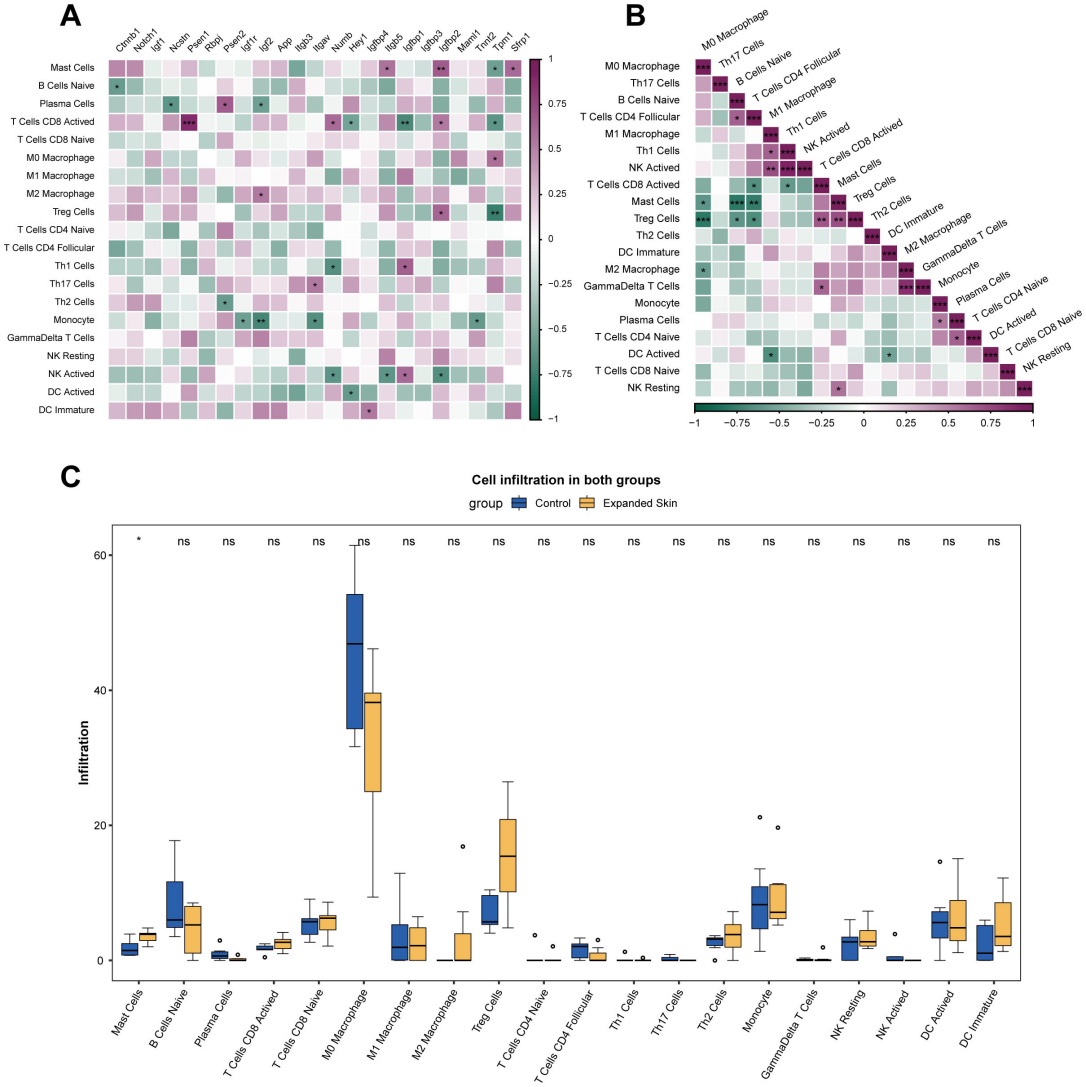
Analysis of immune cell infiltration using ImmuCC based on the integrated dataset. **(A)** Correlation between the infiltration score of 25 immune cells and hub gene expression. Purple indicates positive correlation, while green indicates negative correlation. **(B)** Correlation between infiltration scores of 25 immune cells. Purple indicates positive correlation, while green indicates negative correlation. **(C)** Difference in infiltration fraction of 22 immune cells in expanded group and control. *P < 0.05, **P < 0.01, ***P < 0.001, ns, not significant.

To further analyze the relationship between hub genes and immune cell infiltration, we evaluated 25 immune cell infiltration scores for all samples in the integrated dataset using ImmuCC. The results showed that hub genes, such as *Ctnnb1, Ncstn, Psen1, Psen2, Igf2, Itgav*, and *Numb*, were significantly correlated with the infiltration of various immune cells (*P <* 0.05; **Figure 6A**). In addition, a significant correlation was observed between the infiltration of some immune cells (*P* < 0.05; **Figure 6B**).

The results of the difference analysis of immune cell infiltration between the expanded skin group and the control group showed that most immune cell infiltrations were not significantly different between the two groups (*P* > 0.05; **Figure 6C**). However, number of mast cell (MC) was significantly higher in the expanded group than that in the control group (*P* < 0.05; **Figure 6C**).

### 3.11 Identification of cell type in expanded skin and control

**Figure 7:**
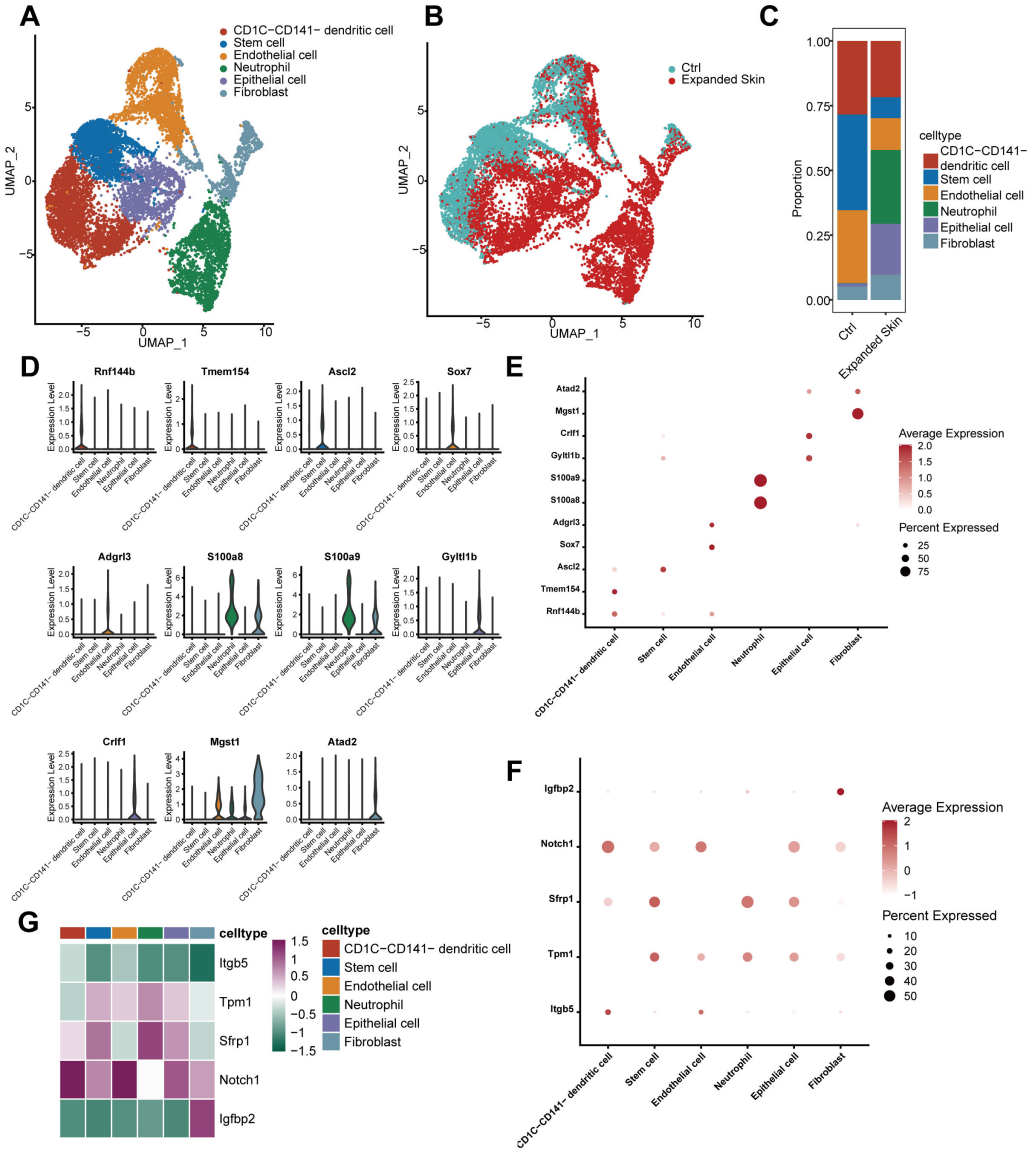
Cell type identification based on GSE146637 dataset. **(A)** UMAP plots of cell clustering. **(B)** Distribution of total cells in expanded skin and control groups. **(C)** Proportion of total cells in expanded skin and controls. **(D)** Violin plots showing the expression levels of characteristic genes for different cell clusters. **(E)** Dot plots showing the expression levels of characteristic genes of different cell clusters. The size of the dots corresponds to the proportion of cells expressing the gene in the cell type. The darker the color, the higher the average expression. **(F)** Dot plots showing the expression levels of diagnostic markers in different cell clusters. The size of the dots corresponds to the proportion of cells expressing the gene in the cell type. The darker the color, the higher the average expression. **(G)** Heat map showing the expression levels of five diagnostic markers in different cell types. Purple indicates high expression, while green indicates low expression. UMAP, Uniform Manifold Approximation and Projection.

Aforementioned results have shown that partial cell infiltration differed significantly between the expanded skin and control groups. Therefore, it is necessary to further explore the heterogeneity and diversity of the cellular composition within these groups. Based on the GSE146637 dataset, we divided 12,263 cells into six groups: CD1C-CD141 dendritic cells, stem cells, endothelial cells, neutrophils, epithelial cells, and fibroblasts (**Figure 7A**). Differences were observed in the distribution of different cell types between the expanded and control groups (**Figure 7B**). Specifically, neutrophils were distributed in the expanded skin group (**Figure 7C**) while stem cells were more abundant in the control group, suggesting that the stem cells in this group may not have differentiated into cell subtypes that promote skin growth.

Considering that the six cell populations specifically expressed unique markers (**Figure 7D**), we could distinguish between different cell types using these markers (**Figure 7E**). We investigated the expression of five diagnostic markers associated with skin growth in different cell types and found that *Igfbp2* was specifically expressed in the fibroblasts (**Figure 7F**). Except for *Itgb5*, all diagnostic markers were clearly expressed in the different cell types (**Figure 7G**).

### 3.12 Intercellular communication in expanded-skin and control groups

**Figure 8:**
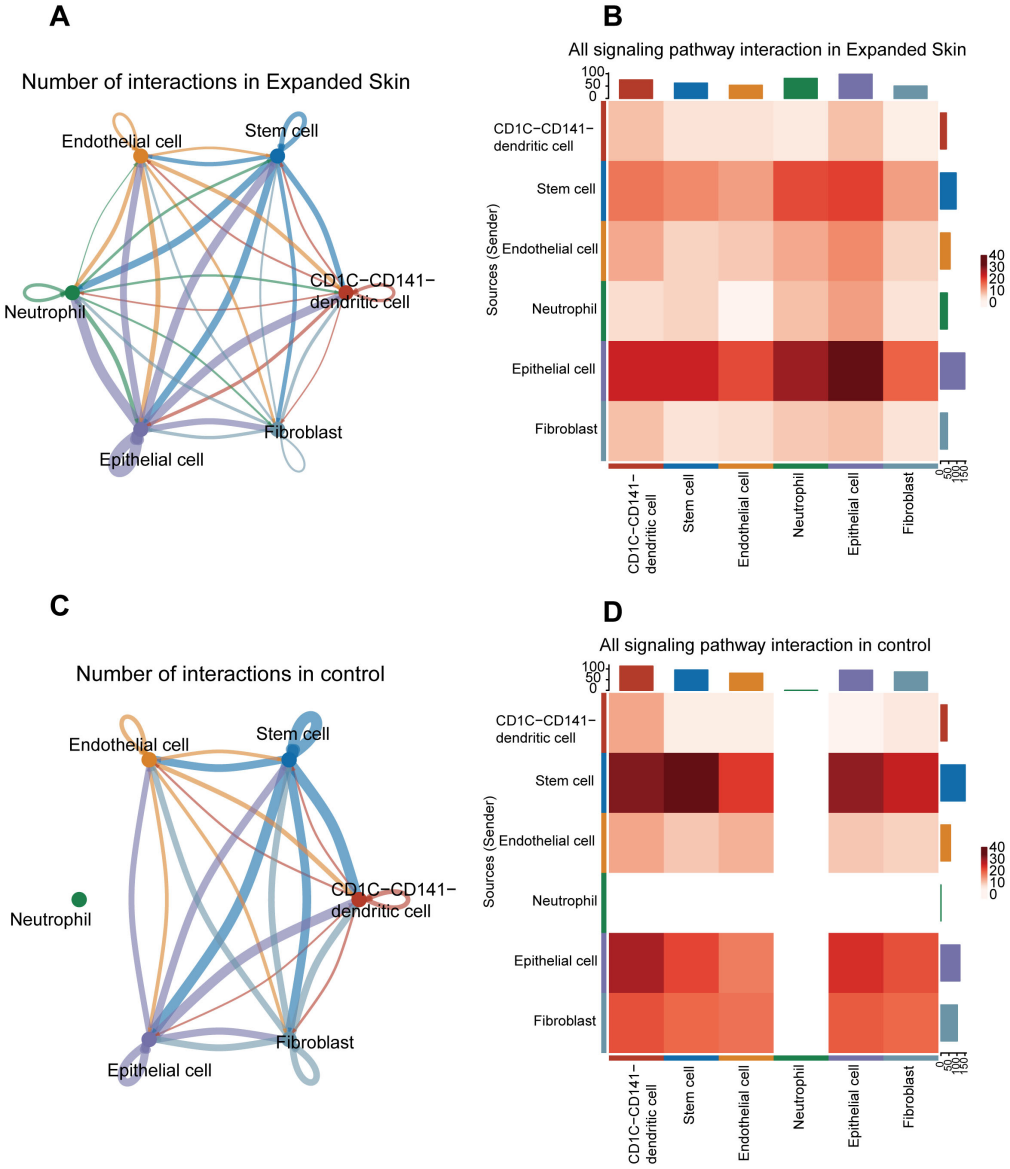
Cell–cell interaction analysis based on the GSE146637 dataset. **(A)** Number of interactions of all cell types in the expanded group. **(B)** Intensity of interactions between different cell types in the expanded group. **(C)** Number of interactions of all cell types in controls. **(D)** Intensity of interaction between different types of cells in control group. The color intensity corresponds to the interaction strength between the different cell types.

Next, we investigated cell-cell interactions between different cell clusters. Based on the expression matrix of the GSE146637 dataset, potential cell-cell interactions were inferred from the ligand-receptor pair data, which were built in CellChat. The number of interactions among epithelial cells, stem cells, and other cell types was higher in the expanded skin group than that in the control group (**Figure 8A**). The strength of the interaction between epithelial cells and epithelial cells, epithelial cells and neutrophils, and epithelial cells and stem cells was the highest (**Figure 8B**).

In the control group, interactions between different cell clusters was shown in **Figure 8C**. The strongest interactions were detected between stem cells, stem cells and epithelial cells, and stem cells and DCs (**Figure 8D**).

### 3.13 Functional differences between cell clusters

GSVA characterized the functional activity of gene sets with specific biological functions in different cell clusters and tissues based on single cell expression matrices. To understand the functional differences of the cell clusters, we selected 50 HALLMARK gene sets and performed GSVA on all cells based on the GSE146637 dataset, followed by limma analysis of the gene sets with functional enrichment differences in the expanded skin and control groups (**Supplementary Data Sheet 5**).

We found that the myogenesis pathway and the Wingless and Int-1 (Wnt)-β-catenin signaling pathway were significantly upregulated in the expanded skin group, while other pathways associated with cell cycle and cell differentiation, such as the G2M checkpoint, E2F targets, and MYC targets, were significantly upregulated in the control group (**Supplementary Data Sheet 2**).

The functional differences between the different cell clusters were further investigated revealing that in the expanded group, most functional activities of endothelial cells, DCs, stem cells, and epithelial cells were upregulated, whereas fibroblasts and neutrophils exhibited downregulation of many functions (**Supplementary Data Sheet 2**). Most functional activities of the different cell types were downregulated in the control group compared to those in the skin growth group (**Supplementary Data Sheet 2**). Pathways associated with cell proliferation and growth, such as Wnt-catenin signaling, the G2M checkpoint, E2F targets, and MYC targets, were also upregulated in multiple cell types in the expanded skin panel (**Supplementary Data Sheet 2**).

### 3.14 Subpopulation identification of stem cells

**Figure 9:**
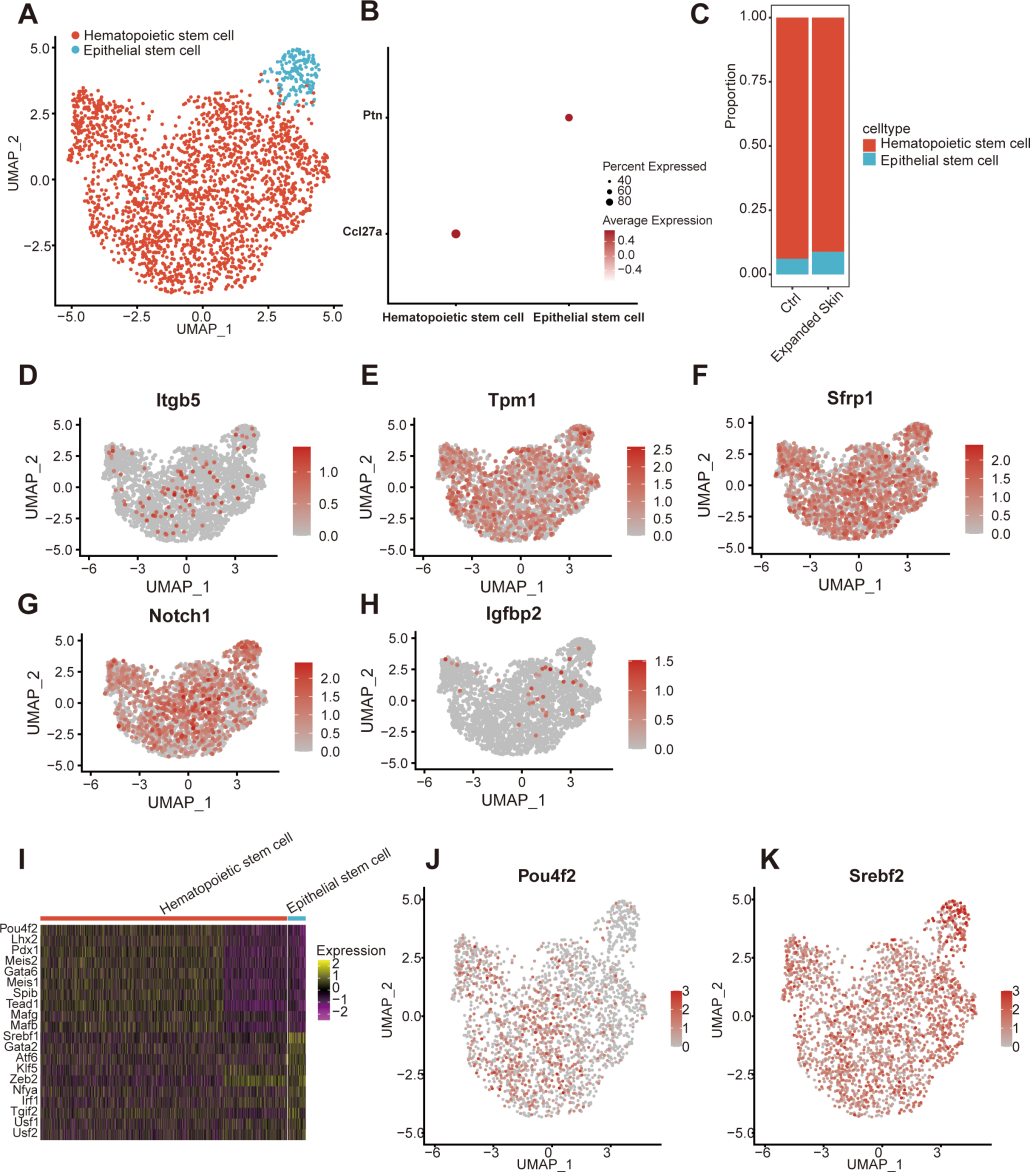
Subpopulation identification of stem cells based on the GSE146637 dataset. **(A)** The 2,334 stem cells were divided into two subpopulations. **(B)** Dot plots showing the expression levels of the marker genes in different subpopulations. **(C)** Content of different cell subsets in the expanded group and control. UMAP plots showing the expression levels of *Itgb5* **(D)**, *Tpm1* **(E)**, *Sfrp1* **(F)**, *Notch1* **(G)**, and *Igfbp2* **(H)**. **(I)** Heat map showing the top 10 TFs with the highest transcriptional activity of different cell subset. Yellow: upregulation of transcriptional activity; purple: downregulation of transcriptional activity. UMAP maps showing transcriptional activity of transcription factor *Pou4f2* **(J)** and *Srebf2* **(K)** in different cell subsets. Red: high transcriptional activity; gray: low transcriptional activity. UMAP, Uniform Manifold Approximation and Projection; TF, transcription factor.

According to intercellular communication analysis, stem cells play a potentially important role in cell interactions of skin growth. Thus, further studies were conducted to classify and annotate the stem cell subsets. Two stem cell subsets were identified: hematopoietic and epidermal stem cells (**Figure 9A**). Among them, hematopoietic stem cells specifically highly expressed the CC motif chemokine ligand 27A marker, while epidermal stem cells expressed pleiotrophin (**Figure 9B**). Furthermore, the proportion of epithelial stem cells was significantly higher in the expanded skin than that in controls (**Figure 9C**).

The expression of five diagnostic markers associated with skin growth was examined in the stem cell subsets. Low expression of *Itgb5* and *Igfbp2*, and gigh expression of *Tpm1, Sfrp1* and *Notch1* was observed in all stem cell subsets (**Figures 9D–H**). Subsequently, we investigated TF activity in all stem cell subsets. Each subpopulation contained a unique TF with upregulated transcriptional activity (**Figure 9I**). The transcriptional activity of Pit-Oct-Unc class 4 homeobox 2 (*Pou4f2*) was upregulated in hematopoietic stem cells, while that of sterol regulatory element binding TF 2 (*Srebp2*) was upregulated in epidermal stem cells (**Figures 9J, K**).

**Figure 10:**
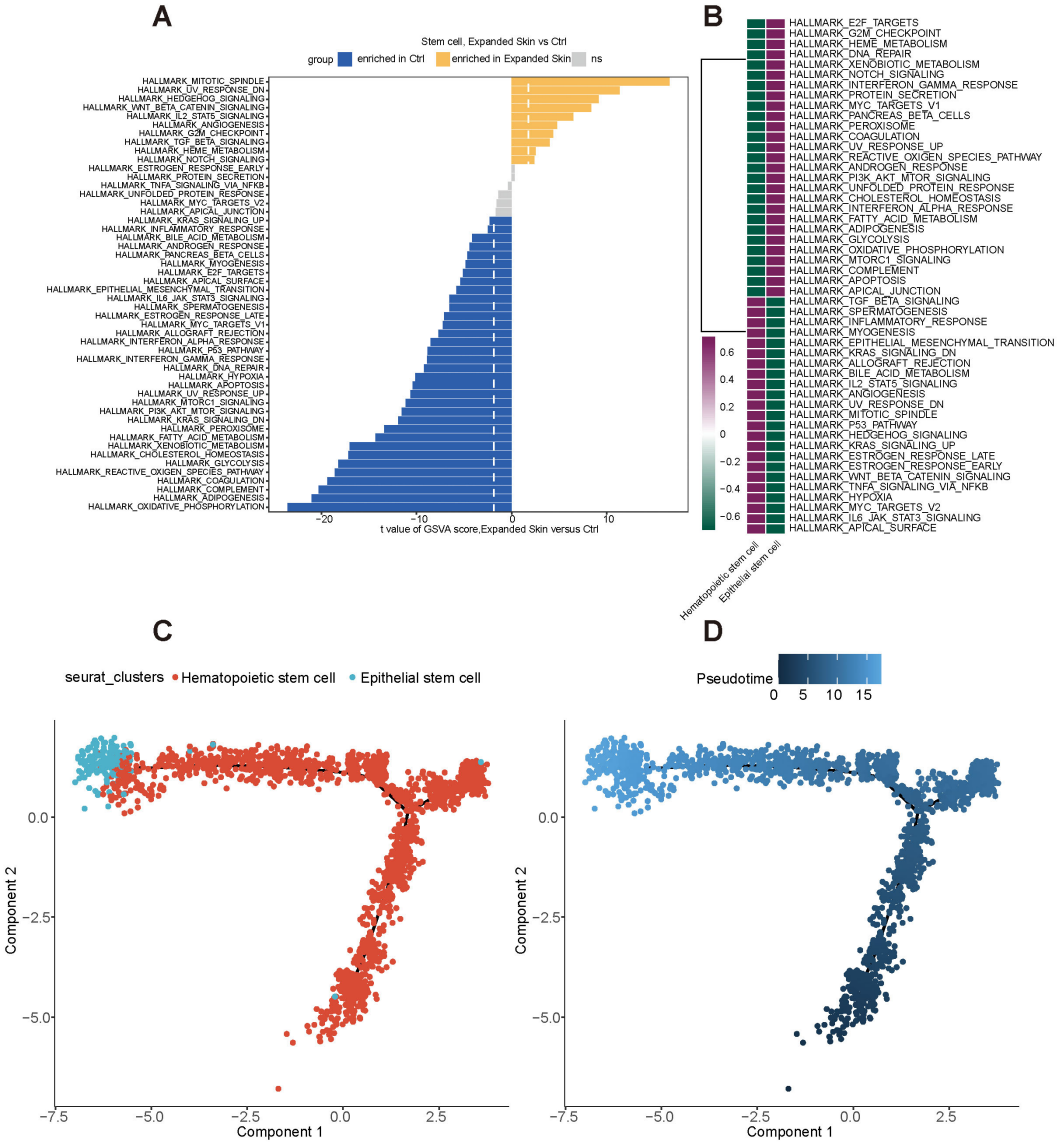
Functional analysis and pseudo-timing analysis of stem cells based on the GSE146637 dataset. **(A)** Differences in the functional activity of 50 HALLMARK gene sets in the expanded skin and control groups. **(B)** Differences in the functional activity of 50 HALLMARK gene sets in different cell subsets. Purple: upregulation of activity; green: downregulation of activity. **(C)** Stem cell subpopulation differentiation trajectory. **(D)** Pseudo-temporal differentiation trajectory showing differentiation of stem cell subsets; dark color indicates the start of differentiation and light color indicates the end of differentiation.

We then performed functional analysis of different cell subtypes and constructed pseudo-temporal trajectories. The GSVA results showed significant differences in the functional activity of most HALLMARK gene sets in stem cells from the expanded skin and normal control groups (**Supplementary Data Sheet 6**). Among them, Wnt-β-catenin signaling pathway was significantly upregulated in stem cells in the expanded skin group (t > 0, *P* < 0.05). Meanwhile, oxidative-phosphorylation, glycolysis, and complement pathways were significantly downregulated in the stem cells of the expanded group (t < 0, *P* < 0.05; **Figure 10A**). However, the functional characteristics of the two stem cell subsets differed significantly, with particular subsets enriched for unique functions (**Figure 10B**).

By constructing pseudotemporal differentiation trajectories of stem cell subsets, we found that hematopoietic stem cells appeared at the beginning of the differentiation trajectory and differentiated into epidermal stem cells (**Figures 10C, D**). These results suggest that the stem cell subsets in the expanded skin group and control group exhibit high heterogeneity and diversity and have unique characteristics in gene expression, functional activity, transcriptional regulation, and differentiation status, which might be the basis for the growth of the expanded mouse skin.

### 3.15 Identification of epithelial cell subpopulations

**Figure 11:**
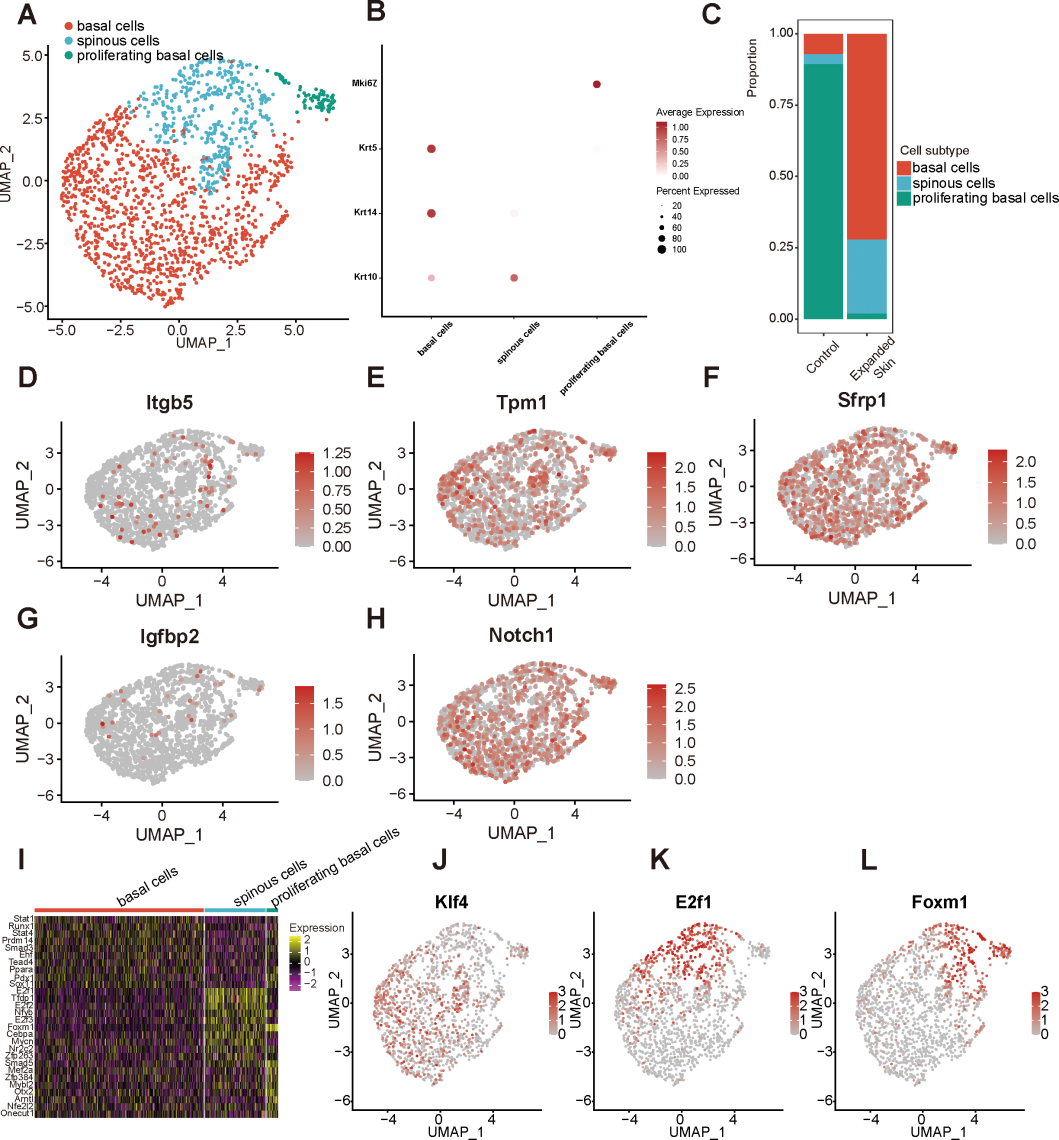
Identification of epithelial cell subpopulations based on the GSE146637 dataset. **(A)** The 1550 epithelial cells were divided into three subpopulations. **(B)** Dot plots showing the expression levels of the marker genes for the different subpopulations. **(C)** Proportion of different cell subsets in the expanded skin and control groups. UMAP map presentation of the expression level of *Itgb5* **(D)**, *Tpm1* **(E)**, *Sfrp1* **(F)**, *Igfbp2* **(G)**, *Notch1* **(H)**. **(I)** Heat map showing the top 10 TFs with the highest transcriptional activity for each cell subset. Yellow: upregulation of transcriptional activity; purple: downregulation of transcriptional activity. UMAP plots of TF transcriptional activity: *Klf4* **(J)**, *E2f1* **(K)**, and *Foxm1* **(L)** in different cell subsets. Red: high transcriptional activity; gray: low transcriptional activity; UMAP, Uniform Manifold Approximation and Projection.

By classifying and annotating cell subsets, we identified three epithelial cell subsets: spinous, proliferating basal, and basal cells (**Figure 11A**). Among them, the basal cell-specific high expression markers keratin 5 (*Krt5*) and *Krt14*, spinus cell-specific high expression marker *Krt10* (**Figure 11B**), and proliferating basal cell-specific high expression marker *Mki67* were identified. Basal cells comprised a significantly high proportion, whereas proliferating basal cells constituted a lower proportion than other epithelial subgroups in the expanded group (**Figure 11C**).

The expression levels of five diagnostic markers were explored in different cell subsets, as shown in **Figures 11D–H**. Among them, *Tpm1* (**Figure 11E**), *Sfrp1* (**Figure 11F**), and *Notch1* (**Figure 11H**) were expressed at high levels in all subgroups. Subsequently, the TF activity was investigated in all cell subsets, revealing that each subpopulation contained a unique TF with upregulated transcriptional activity (**Figure 11I**). Among them, the transcriptional activity of Krüppel-like factor 4 (*Klf4*) was upregulated in basal cells, that of *E2f1* was upregulated in spinous cells, and that of forkhead box M1 (*Foxm1*) was upregulated in spinous and proliferating basal cells (**Figures 11J–L**).

**Figure 12:**
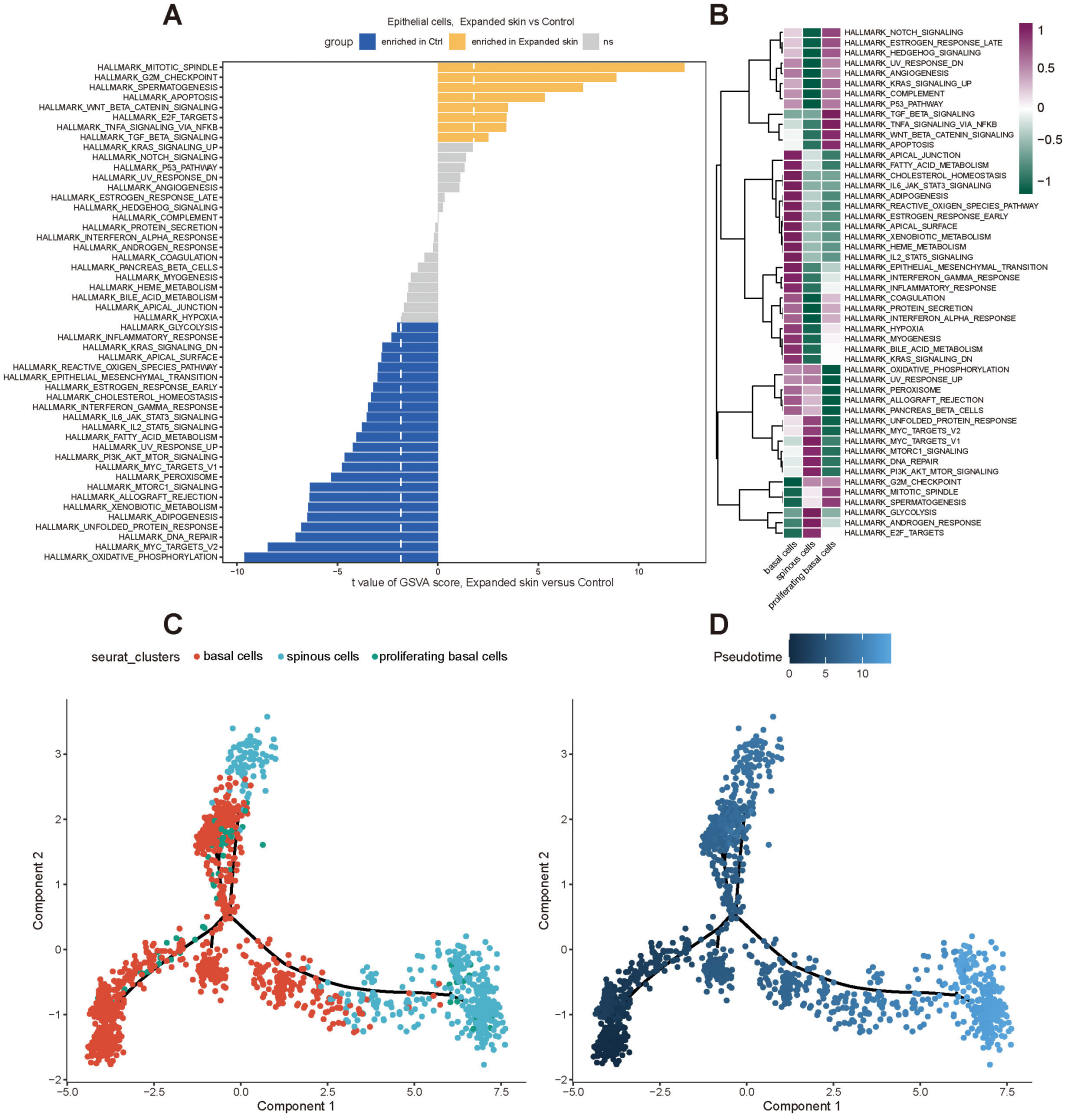
Functional analysis and pseudo-temporal analysis of the epithelial cells based on the GSE146637 dataset. **(A)** Differences in functional activity of 50 HALLMARK gene sets in expanded skin and control groups. **(B)** Differences in functional activity of 50 HALLMARK gene sets in different cell subsets. Purple: upregulation of functional activity; green: downregulation of functional activity. **(C)** Epithelial cell subpopulation differentiation trajectory. **(D)** Pseudo-temporal display of differentiation trajectories of epithelial cell subsets; dark color indicates the start of differentiation and light color indicates the end of differentiation.

Subsequently, we performed functional analysis of different cell subtypes and constructed pseudo-temporal trajectories. The GSVA results showed significant differences in the functional activity of most HALLMARK gene sets in epithelial cells from the expanded skin and control groups (see **Supplementary Data Sheet 7**). Among them, the G2M checkpoint was significantly upregulated in the expanded group (t > 0, *P* < 0.05), and MYC targets V2 and oxidative-phosphorylation were significantly upregulated in the control group (t < 0, *P* < 0.05; **Figure 12A**). However, the functional characteristics of different cell subtypes differed significantly, suggesting that they had unique roles in the different groups (**Figure 12B**).

By constructing pseudo-temporal differentiation trajectories of epithelial cell subsets, we also found that basal cells appeared at the beginning of the differentiation trajectories and differentiated into spinous cells after the intermediate cell state of proliferating basal cells (**Figures 12C, D**).

### 3.16 Subpopulation identification of fibroblasts

**Figure 13:**
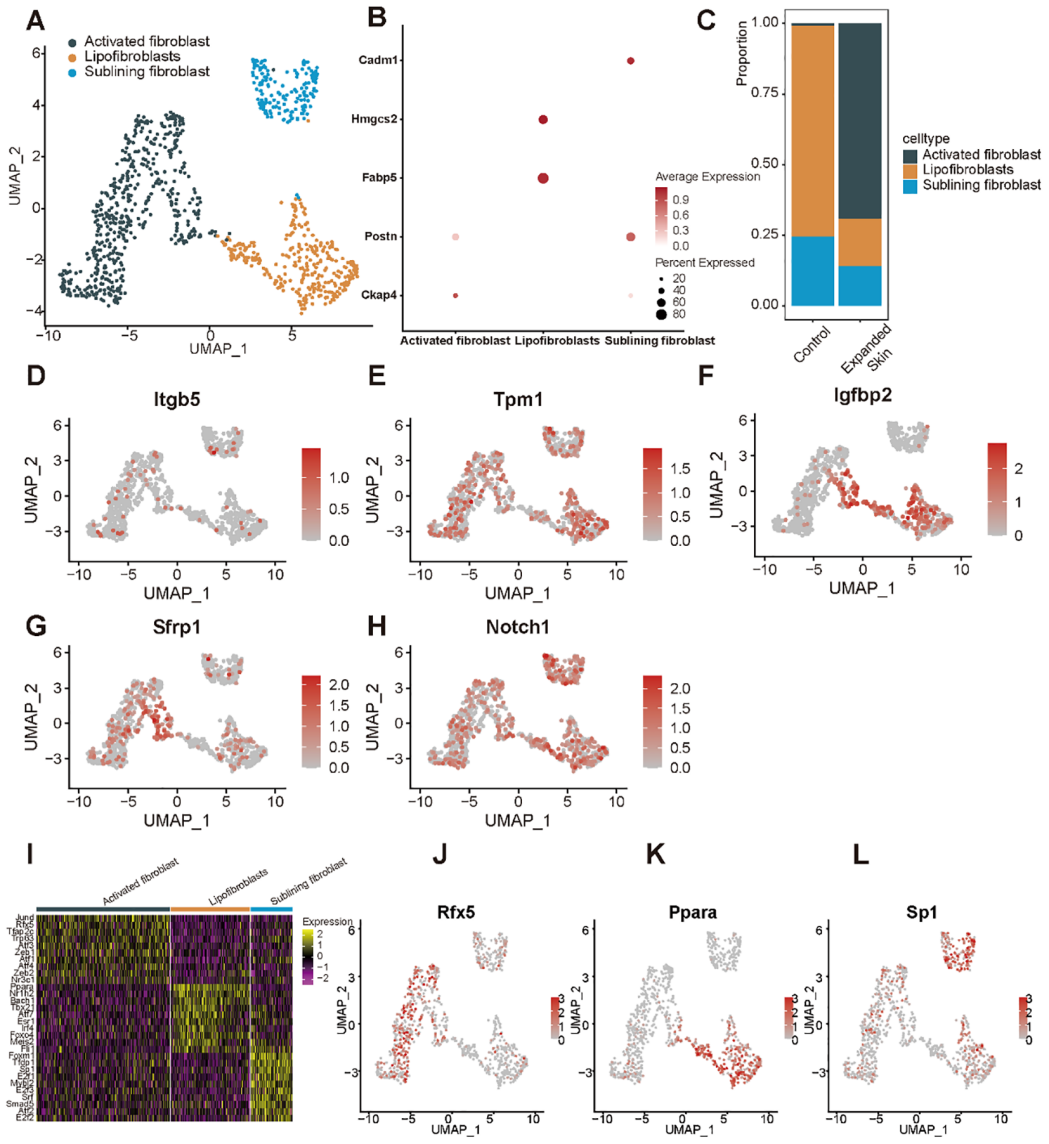
Subpopulation identification of fibroblasts based on the GSE146637 dataset. **(A)** The 982 fibroblasts were divided into three subpopulations. **(B)** Dot plots showing expression of the marker genes for the different subpopulations. **(C)** Content of different cell subsets in expanded skin and control groups. UMAP plots showing the expression levels of *Itgb5* **(D)**, *Tpm1* **(E)**, *Igfbp2* **(F)**, *Sfrp1* **(G)**, and *Notch*1 **(H)** in different cell subsets. **(I)** Heat map showing the top 10 TFs with the highest transcriptional activity of each cell subset. Yellow: upregulation of transcriptional activity; purple: downregulation of transcriptional activity. UMAP maps showing transcriptional activity of transcription factors *Rfx5* **(J)**, *Ppara* **(K)**, and *Sp1* **(L)** in different cell subsets. Red: high transcriptional activity; gray: low transcriptional activity.

By classifying and annotating the cell subsets, we identified three subpopulations of fibroblasts: activated, lipofibroblasts, and sublining fibroblasts (**Figure 13A**). Among them, the activated fibroblasts highly expressed specific marker cytoskeleton-associated protein 4 (*Ckap4*), lipofibroblasts specifically highly expressed markers fatty acid binding protein 5 (*Fabp5*), and 3-hydroxy-3-methylglutaryl-CoA synthase 2 (*Hmgcs2*), and sublining fibroblast-specific highly expressed markers *Postn* and cell adhesion molecule 1 (*Cadm1*) (**Figure 13B**). The number of lipofibroblasts was significantly higher in the control group while activated fibroblasts were significantly more abundant than other subpopulations in the expanded skin group (**Figure 13C**).

We then explored the expression levels of the five diagnostic markers in the different cell subsets. The expression of five markers in fibroblasts was shown in **Figures 13D–H**. Subsequently, we investigated TF activity in all cell subsets and found that each subpopulation had a unique TF with upregulated transcriptional activity (**Figure 13I**). The transcriptional activity of regulatory factor X5 (*Rfx5*) was upregulated in the activated fibroblasts (**Figure 13J**), while that of peroxisome proliferator-activated receptor alpha (*Ppara*) was upregulated in the lipofibroblasts (**Figure 13K**), and the expression of the specificity protein 1 (*Sp1*) was upregulated in the sublining fibroblasts (**Figure 13L**).

**Figure 14:**
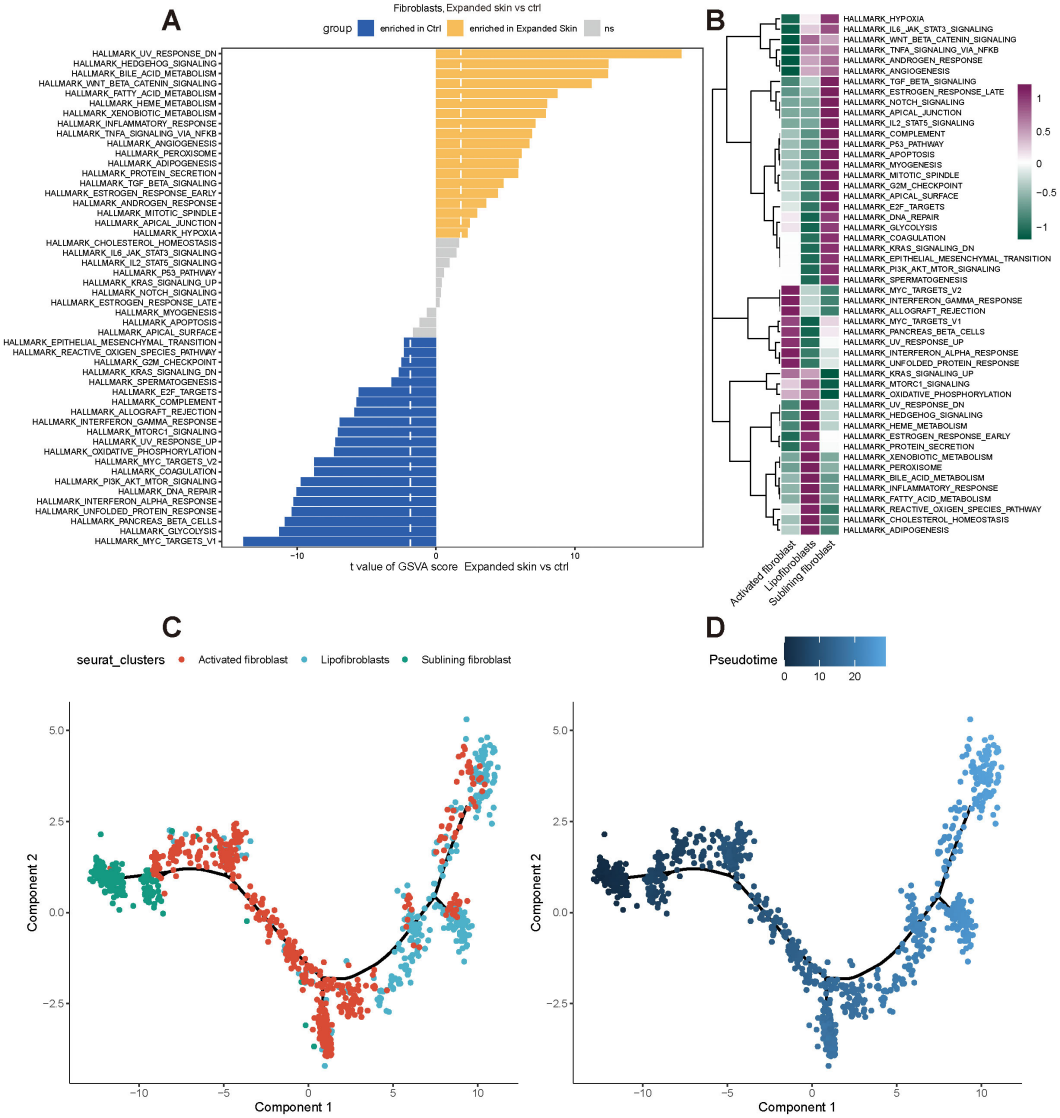
Functional analysis and pseudo-temporal analysis of fibroblasts based on GSE146637 dataset. **(A)** Differences in functional activity of 50 HALLMARK gene sets in expanded skin group and control group. **(B)** Differences in functional activity of 50 HALLMARK gene sets in different cell subsets. Purple indicates upregulation of functional activity, green indicates downregulation of functional activity. **(C)** Fibroblast subpopulation differentiation trajectory. **(D)** Pseudo-temporal display of differentiation trajectories of subpopulations of fibroblasts; dark color indicates the start of differentiation and light color indicates the end of differentiation.

Subsequently, we performed functional analysis of different cell subtypes and constructed pseudo-temporal trajectories. The GSVA results showed significant differences in the functional activity of most HALLMARK gene sets in fibroblasts between the expanded skin and control groups (**Supplementary Data Sheet 8**). Among them, Wnt-β-catenin signaling was significantly upregulated in fibroblasts in the expanded group (t > 0, *P* < 0.05), and MYC targets-V1 and glycolysis were significantly up-regulated in fibroblasts in the control group (t < 0, *P* < 0.05; **Figure 14A**). However, the functional characteristics of different cell subsets differed significantly, with different subsets enriched for unique functions (**Figure 14B**).

By constructing pseudo-temporal differentiation trajectories of fibroblast subsets, we also found that sublinear fibroblasts appeared at the beginning of the differentiation trajectory, and after intermediate cell state activated fibroblasts, differentiated into lipofibroblasts (**Figures 14C, D**).

### 3.17 Identification of endothelial cell subpopulations

**Figure 15:**
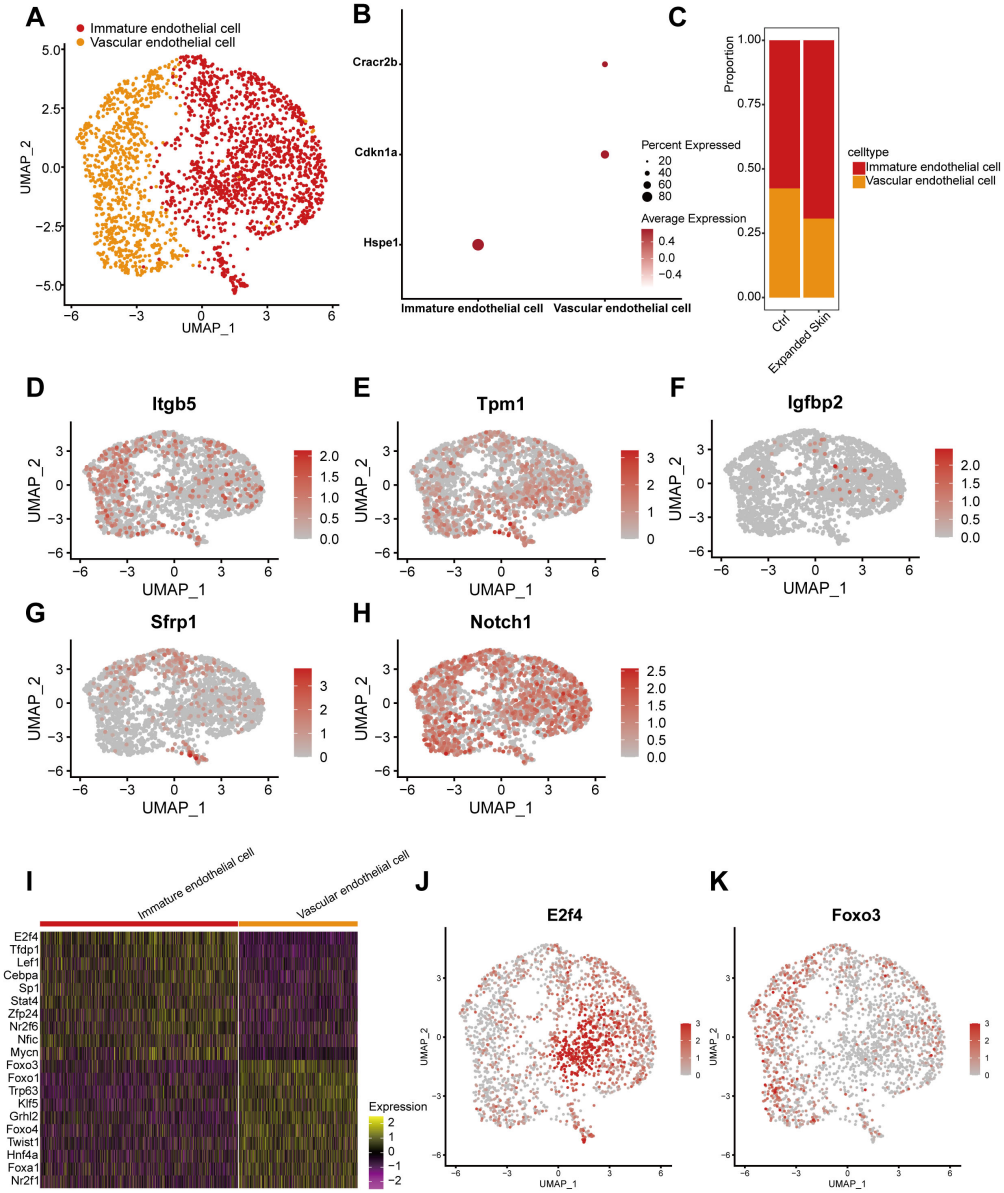
Subpopulation identification of endothelial cells based on the GSE146637 dataset. **(A)** The 2,244 endothelial cells were divided into two subpopulations. **(B)** Dot plots showing the expression levels of the marker genes for the different subpopulations. **(C)** Proportion of different cell subsets in the expanded skin and control groups. UMAP plots showing the expression levels of *Itgb5* **(D)**, *Tpm1* **(E)**, *Igfbp2* **(F)**, *Sfrp1* **(G)**, and *Notch1* **(H)** in different cell subsets. **(I)** Heat map showing the top 10 TFs with the highest transcriptional activity for each cell subset. Yellow: upregulation of transcriptional activity; purple: downregulation of transcriptional activity. UMAP maps showing the transcriptional activity of transcription factors *E2f4* **(J)** and *Foxo3* **(K)** in different cell subsets. Red: high transcriptional activity; gray: low transcriptional activity.

By classifying and annotating cell subsets, we identified two subsets, immature endothelial cells and vascular endothelial cells (**Figure 15A**). Among them, heat shock protein family E member 1 (*Hspe1*) was highly expressed in immature endothelial cells, and the markers cyclin-dependent kinase inhibitor 1A (*Cdkn1a*) and calcium release-activated channel regulator 2B (*Cracr2b*) were highly expressed in vascular endothelial cells (**Figure 15B**). The proportion of immature endothelial cells was higher in the expanded group than that in the control group (**Figure 15C**).

Additionally, the expression of the five diagnostic markers was evaluated in the different cell subsets, as shown in **Figures 15D–H**. Subsequently, we investigated TF activity and found that each subpopulation contained a unique TF with upregulated transcriptional activity (**Figure 15I**). Among these, the transcriptional activity of *E2f4* (**Figure 15J**) was upregulated in immature endothelial cells, while that of forkhead box O3 (*Foxo3*) was upregulated in vascular endothelial cells (**Figure 15K**).

**Figure 16:**
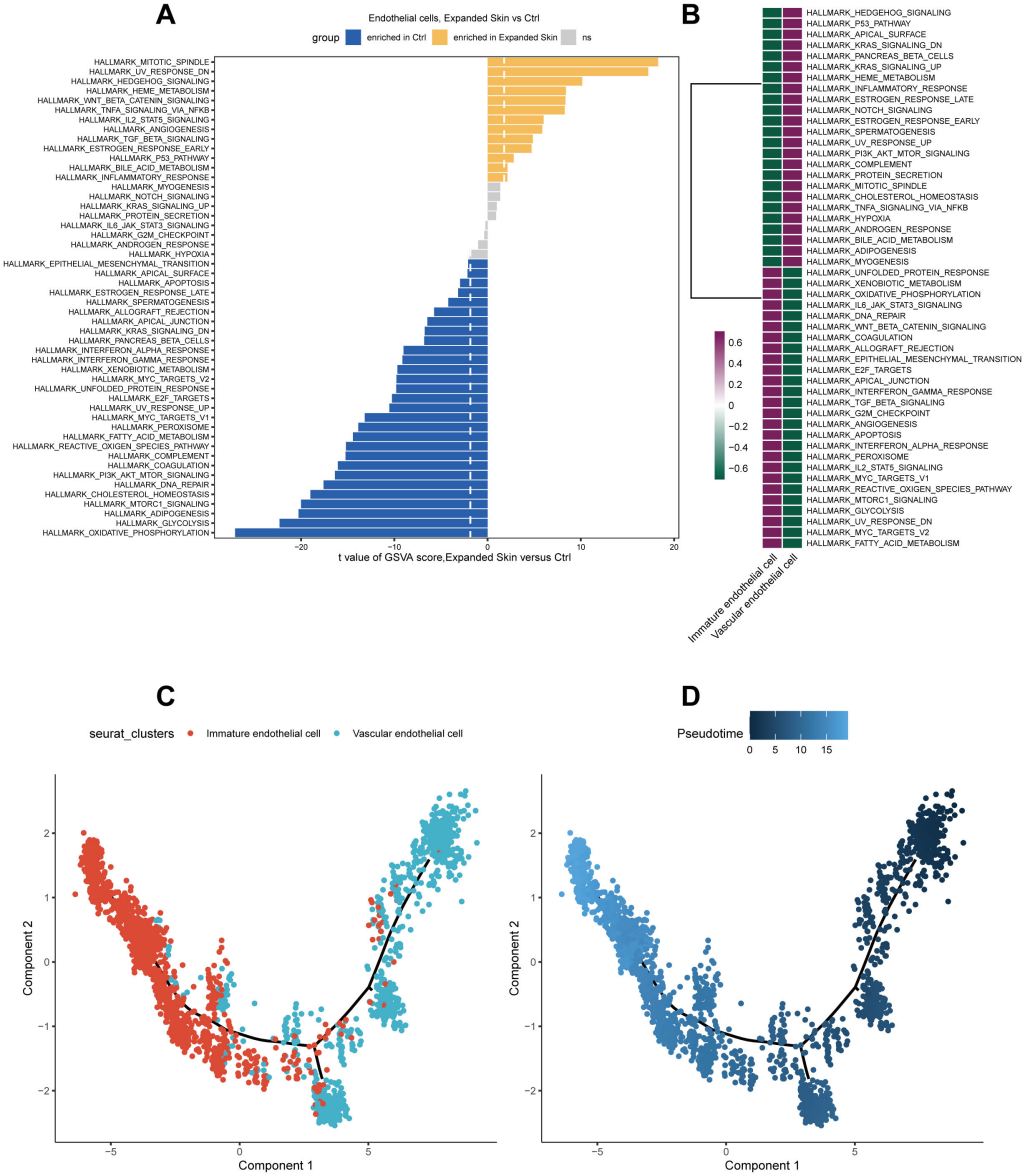
Functional analysis and pseudo-temporal analysis of endothelial cells based on the GSE146637 dataset. **(A)** Differences in the functional activity of 50 HALLMARK gene sets in the expanded and control groups. **(B)** Differences in the functional activity of 50 HALLMARK gene sets in different cell subsets. Purple: upregulation of functional activity; green: downregulation of functional activity. **(C)** Endothelial cell subpopulation differentiation trajectory. **(D)** Pseudo-temporal display of differentiation trajectories of endothelial cell subsets; dark color indicates the start of differentiation and light color indicates the end of differentiation.

Subsequently, we performed functional analysis of different cell subtypes and constructed pseudo-temporal trajectories. GSVA results showed that the functional activity of most HALLMARK gene sets in endothelial cells from the expanded skin and control groups differed significantly (see **Supplementary Data Sheet 1**). Among them, TNF-α signaling via NF-κB was significantly upregulated (t > 0, *P* < 0.05), and oxidative phosphorylation and glycolysis were significantly downregulated in endothelial cells in the expanded group (t < 0, *P* < 0.05; **Figure 16A**). However, the functional characteristics of different cell subsets differed significantly, with different subsets enriched for unique functions (**Figure 16B**).

By constructing a pseudotemporal differentiation trajectory of the endothelial cell subsets, we found that vascular endothelial cells appeared at the beginning of the differentiation trajectory and differentiated into immature endothelial cells (**Figures 16C, D**). These results suggest that stem cell, epithelial cell, fibroblast, and endothelial cell subsets in the expanded and control groups exhibited considerable heterogeneity and diversity.

## 4 Discussion

In the current study, we constructed a diagnostic model for EMTRDEGs based on an integrated dataset using a random forest analysis and identified the top five genes (*Itgb5, Tpm1, Sfrp1, Notch1*, and *Igfbp2*) with the greatest impact on the model’s predictive capacity. A PPI network of these five genes was constructed using the STRING database to identify hub genes associated with skin growth. We selected the top 20 genes with the highest network connectivity (excluding genes not included in the expression matrix of the integrated dataset) and 5 diagnostic markers associated with skin growth in the expanded tissue. Among these, *Notch1, Ncstn, Ctnnb1*, and *Psen1* had higher connectivity than the other genes.

To further evaluate the interactions between hub genes and TFs, we constructed a TF interaction network with the hub genes and found that *Itgb3, Notch1*, and *Rbpj* were the most connected and may have the strongest interactive relationships with TFs. KEGG and GO results showed that the hub genes were primarily enriched in the Notch, PI3K-AKT, insulin-like growth factor receptor, and thyroid hormone signaling pathways, which are important in cell proliferation, ECM remodeling, and cell growth regulation. Upregulated *Ncstn* expression reportedly promotes hepatocellular carcinoma cell growth and metastasis via β-catenin activation in a Notch1-dependent manner (35), while its mutation disrupts the development of hair follicles (36). Meanwhile, *Psen1* functions as a causative factor for early onset Alzheimer’s disease, and as a part of γ-secretase, impacts Notch signaling and β-cadherin processing (37).

In this study, we first analyzed the functions of the hub genes in skin growth under mechanical stretching. The results of the immune infiltration analysis showed that *Ctnnb1, Psen1*, and *Igf2* were significantly correlated with the infiltration of various immune cells, including a positive correlation between MCs and *Itgb5* and *Igfbp2*, M0 macrophages and *Tpm1*, and M2 macrophages and *Igf2*. Moreover, a negative correlation was observed between MCs and *Tpm1* and T regulatory (Treg) cells and *Tpm1*. Number of MC was significantly higher in the expanded skin group than that in the control group. In addition, a significant correlation was observed between the infiltration of various immune cells, including a positive correlation between MCs and Tregs and negative correlations between Tregs and M0 macrophages, and MCs and M0 macrophages. To further explore the heterogeneity and diversity of the cellular composition, a single-cell sequencing dataset analysis was conducted. Based on the cell-cell interaction analysis, the number of interactions between epithelial cells and stem cells with other cell types was higher in the expanded skin group than that in the control group, demonstrating the importance of stem cells and epithelial cells in expanded skin. Stem cells retain remarkable plasticity and self-renewal capabilities, enabling them to replenish expanded skin and maintain skin homeostasis.

MCs that are widely distributed around the vessels and sensory nerves of the skin and visceral mucosa secrete myriad cytokines that modulate angiogenesis and fibrosis and participate in immune regulation by inducing vasodilation, promoting vascular permeability, and recruiting inflammatory cells (38). MCs and the mediators derived from MCs participate in angiogenesis and all stages of skin regeneration during wound healing. Furthermore, mechanical stretch promotes MC degranulation, which activates the TGF-β1 pathway for the EMT process and fibroblast activation (39). Thus, MCs may play a vital role in the immune response to mechanical stretching in the expanded skin model, which is consistent with the immune infiltration analysis performed in this study. Recruited immune cells, such as DCs, macrophages, and T cells, interact with MCs. MC-derived soluble proteins promote DC activation; direct synaptic connections between MCs and DCs exchange internalized antigens that are presented to T cells to induce activation (38). MC-derived IL-10 can reduce DC migration and activation and enhance the ability of DCs to reduce T-cell proliferation and cytokine production. Furthermore, IL-10 suppresses proinflammatory cytokine production by monocytes, macrophages, and neutrophils (40). This study suggested a potential upregulation of IL-10 expression of MCs in expanded skin. Nonetheless, additional experiments are required to validate this observation. Moreover, TNF-α and IL-6 derived from MCs are involved in local recruitment of neutrophils and macrophage activation (41). In addition, TGF-β derived from MCs participates in Treg generation. IL-10 and TGF-β secreted by Tregs may reciprocally influence MC function (42). Collectively, these data may explain the negative correlation between MCs and macrophages, and the positive correlation between MCs and Tregs in this study.

Intriguingly, macrophages, as a vital immune cell group mediating tissue regeneration in expanded skin, exhibited minor differences in tissue infiltration between the expanded skin and control groups. The function of macrophages varies significantly depending on the stretch intensity and temporal and spatial distribution (39, 43). Thus, the function, temporal and spatial variation of macrophages during skin expansion and their relationship with the surrounding cells warrants further exploration.

The diagnostic marker *Igfbp2* was specifically expressed in fibroblasts. Except for *Itgb5* and *Igfbp*2, all diagnostic markers were clearly expressed in the different cell populations. Further analysis showed that in the expanded skin group, most functional activities of endothelial cells, DCs, stem cells, and epithelial cells were upregulated, while fibroblasts and neutrophils exhibited downregulation of many functions. We also identified hematopoietic and epidermal stem cells among the stem cell subsets and found that the amount of hematopoietic stem cells was significantly high in both expanded and control groups. Meanwhile, low expression of *Itgb5* and *Igfbp2* and high expression of *Tpm1, Sfrp1*, and *Notch1* were observed in the stem cell subsets. The GSVA results further revealed that the Wnt-β-catenin signaling pathway was significantly upregulated in stem cells in the expanded skin group. We also identified three epithelial cell subsets: spinous, proliferating basal, and basal cells. The proportions of basal and spinous cells were significantly higher in the expanded group than that in the control group. *Itgb5* and *Igfbp2* were expressed at low levels in all epithelial cell subgroups, whereas *Tpm1, Sfrp1*, and *Notch1* were expressed at high levels in all subgroups. By constructing pseudotemporal differentiation trajectories of epithelial cell subsets, we found that basal cells appeared at the beginning of the differentiation trajectories and differentiated into spinous cells following an intermediate state as proliferating basal cells. The GSVA results further revealed that the Wnt-β-catenin signaling pathway was significantly upregulated in the fibroblasts of the expanded group. Moreover, two endothelial cell subsets, immature and vascular endothelial cells, were identified in the data set. *Igfbp2* and *Sfrp1* were downregulated in all subsets, whereas *Itgb5, Tpm1*, and *Notch1* were upregulated across all endothelial cell subsets. GSVA results showed that TNFα via the NF-κB signaling pathway was significantly upregulated in endothelial cells in the expanded skin group. According to the single-cell data, *Tpm1, Sfrp1*, and *Notch1*, which were highly expressed in all subgroups of epithelial cells and stem cells, may be vital indicators of skin growth in the skin expansion model.

Skin regeneration and the constant turnover of cells in the expanded skin model upon mechanical stretching necessitate the renewal and differentiation of stem cells. Interactions between stem cells and their niches vary during exposure to different environments (44). In particular, the epidermis acts as a protective barrier, and stem cells located in the basal layer maintain epidermal renewal and initiate skin regeneration in the expanded skin model. Consistent with previous studies, increased stem cell renewal and basal populations were observed in the expanded skin group. However, in contrast to a previous study by Sun et al. (4) the renewal of proliferative cells showed an increasing trend, and spinous cells as non-proliferative cells also increased, which may contribute to the differences between rapid skin expansion models and clinical moderate constant-volume skin expansion paradigm processes. Meanwhile, dermal fibroblasts are a rich source of mitogens that facilitate the proliferation of epidermal cells and may account for the upregulation of TFs, such as E2F1 and KLF4, in the epithelial subgroups (45). Capillary networks supply the skin with oxygen, nutrients, and hormones, and facilitate immune cell infiltration upon stimulations (46). Immature endothelial cells, which participate in postnatal neovascularization, were upregulated in the expanded skin group, indicating that neovascularization is necessary for skin growth in the early stage of skin expansion (47). The increased expression of the TNFα NF-κB signaling pathway may further promote the EMT process to generate a positive feedback loop during skin growth under mechanical stretch stimulation (6). A previous histological analysis also found angiogenesis in expanded tissues (48). Endothelial cells also function as a barrier between resident dermal cells and circulating immune cells, while cytokines may have essential immunomodulatory roles in expanded tissue, which requires further exploration.

The hub gene, *Notch1*, as part of Notch signaling, activates epidermal stratification and participates in the EMT and Wnt signaling pathways (49). In the Wnt pathway, Notch1 represses Wnt signaling and restricts its activation to the basal layer (50). Hence, Notch1 may function as a factor to balance epidermal proliferation and differentiation in expanded skin. Meanwhile, *Tpm1*, which promotes cell movement during wound healing, enables actin filament-binding activity and functions as an immune-related molecule (51).

*Ctnnb1* had higher connectivity than the other hub genes, and *Rbpj* exhibited strong connections with TFs. Ctnnb1, a component of adherent junctions in epithelial cells, also functions as a key signaling molecule in canonical Wnt signaling, which could explain its high connectivity with other hub genes in the expanded skin group (52). Further, RBP-Jκ encoded by *Rbpj* is endowed with intrinsic transcription repressive function and can be converted by Notch and other cofactors into an activator. Upregulated RBP-Jκ expression enhances keratinocyte proliferation and is inversely correlated with keratinocyte differentiation. Hence, the relationship between RBP-Jκ and Notch may indicate a balance between differentiation and proliferation in the epithelial tissue of expanded skin, which requires further investigation (53). Tpm1 elicits inflammation by phosphorylating protein kinase and regulating NF-κB signaling (54) and participates in TGF-β-induced actin fiber and matrix adhesion (55), suggesting that Tpm1 may modulate cell movement and the immune response in expanded skin. Furthermore, Sfrp1 enables Wnt protein binding and acts as an antagonist of the Wnt signaling pathway (56). In this study, all stem cell and fibroblast groups showed upregulated Wnt signaling and *Sfrp1* expression, and the epithelial groups also exhibited upregulated *Sfrp1* expression. Proliferating basal cells also upregulated Wnt, while other epithelial subgroups did not, implying that SFRP1 functions as an inhibitor of Wnt signaling in these cells and may have a dominant role in Wnt signaling-induced epithelial differentiation after differentiation into spinous cells.

Although the results of this study could inspire further exploration of expanded skin and help identify skin growth in expanded models, several limitations should be considered. First, based on the analysis of chip and single-cell data acquired from a rapid-expansion mouse model, there may be differences between experimental and clinical expansion treatments at the gene and protein levels. Second, the rapid expansion model reflects the early response of skin expansion, while the late stage of expanded skin under long-term serial stretching requires further exploration. This study suggests the potential correlation between the expression of hub genes and the outcome of skin expansion, and further investigations are needed to verify the correlation in order to improve the outcomes of expansion therapies.

In conclusion, our study provides a reference for predicting early-stage skin growth in a skin expansion model and implicates the significance of immune cells, in particular, that of MCs. According to the expression of hub genes and cell-cell relationships, stem cell and epithelial cell groups showed higher expression of hub genes and cell-cell interactions than fibroblasts and endothelial cells, indicating that stem cells and epithelial cells may have predominant roles in skin regeneration under mechanical expansion. Furthermore, the hub genes *Notch1, Tpm1*, and *Sfrp1* serve as key effectors in modulating epithelial cells, fibroblast, stem cells, and endothelial cells in expanded skin, with strong association with the Notch and Wnt signaling pathways, suggesting potential therapeutic strategies for skin growth enhancement under mechanical expansion.

## Data Availability

The original contributions presented in the study are included in the article/**Supplementary Material**. Further inquiries can be directed to the corresponding authors.
