# Supplementary material for: Comprehensive analysis of skin growth-related hub genes and microenvironment characterization in a mouse expanded skin model
Source: Front Immunol. 2024 Dec 5;15:1306353. doi: 10.3389/fimmu.2024.1306353 (PMC11655345; doi:10.3389/fimmu.2024.1306353)
Supplement: Supplementary file 1 [file DataSheet1.pdf]

Supplementary 1. Construction of skin growth molecular subtypes

Consensus clustering is a resampling-based algorithm that identifies each member and its subgroup number, and verifies the plausibility of the cluster. Consistent clustering involves multiple iterations over subsamples of a dataset and provides an indicator of clustering stability and parameter decisions by inducing sampling variability with subsampling. The consensus clustering using the R package ConsensusClusterPlus (version 1.58.0) identified different subtypes of expanded groups based on hub genes[1].

2.1 Two distinct skin growth subtypes identified by hub genes

Using the ConsensusClusterPlus package in R software, based on 23 hub genes, according to the cumulative distribution curve (Supplementary Figure 5A) of different cluster numbers (k values) and the change of area under the cumulative distributive function (CDF) curve for different k values compared with k-1 value (Supplementary Figure 5B), finally two skin growth subtypes (Cluster 1 and Cluster 2) were identified by consensus clustering method (Supplementary Figure 5C). Cluster 1 contains three skin growth panel samples and Cluster 2 contains four skin growth panel samples.

2. 2 Immune cell infiltration analysis of skin growth subtypes

Next, we investigated the differences in immune cell infiltration and hub gene expression between the two different skin growth subtypes based on the integrated dataset. Immune infiltration analysis showed that there was no significant correlation between most immune cell infiltrates ($p > 0.05$, Supplementary Figure 6A), whereas resting natural killer (NK) cells and T-helper 1 (Th1) cells, activated dendritic cells (DCs), and CD4 naïve T cells, CD8 Activated and mast cells, Th17 cells and M2 macrophages, activated NK cells and M2 macrophages, activated NK cells and Th17 cells, monocytes and plasma cells, and monocytes and M1 macrophages had significant positive correlations ($p < 0.05$, Supplementary Figure 6A). Subsequently, the differences in immune infiltration between the two subtypes were compared, and it was found that only the infiltration of M1 macrophages was significantly different between the two groups, and the degree of infiltration was higher in Cluster 1 ($p < 0.05$; Supplementary Figure 6B). In addition, hub genes such as Notch1, Igf1, Ncstn, Psen1, Psen2, Igf1r, and Itgav were significantly correlated with the infiltration of various immune cells ($p < 0.05$; Supplementary Figure 6C). Igf1r, Igf2, Itgav, and Tnnt2 were upregulated in Cluster 2 ($p < 0.05$; Supplementary Figure 6D).

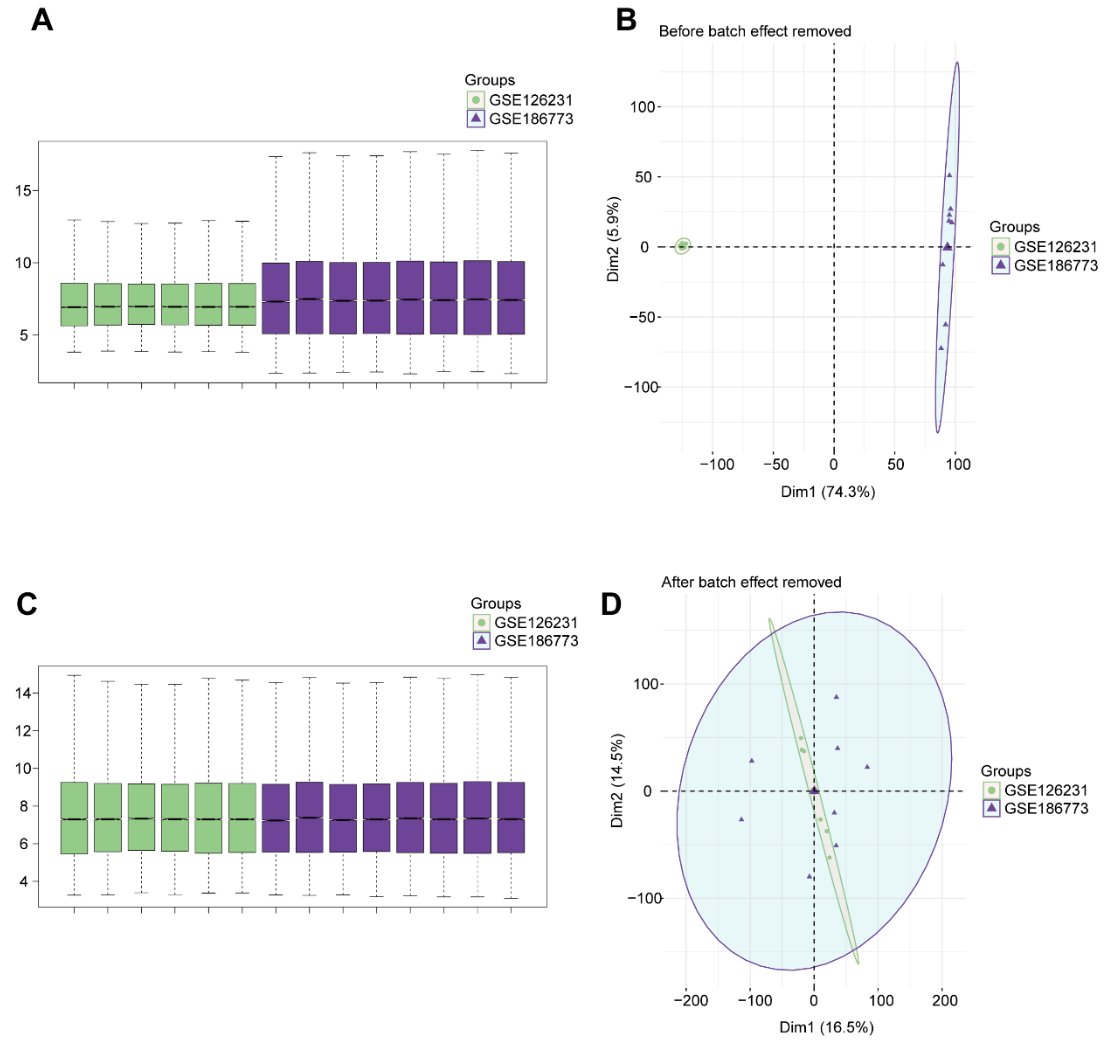

Supplementary Figure 1. Integration of de-batch effects based on GSE126231 and GSE186773 datasets. A. Boxplot of the distribution of samples from the GSE126231 and GSE186773 datasets, respectively, before batch effect removal. B. PCA plots of samples from GSE126231 and GSE186773 datasets before batch effect removal; C. Box plots of sample distribution from GSE126231 and GSE186773 datasets, respectively, after batch effect removal; D. PCA plots from GSE126231 and GSE186773 datasets, respectively, after batch effect removal. PCA, Principal Component Analysis. The results of the distribution box plot and PCA plot show that the batch effect of the samples in the data sets is basically eliminated after the de-batch process.

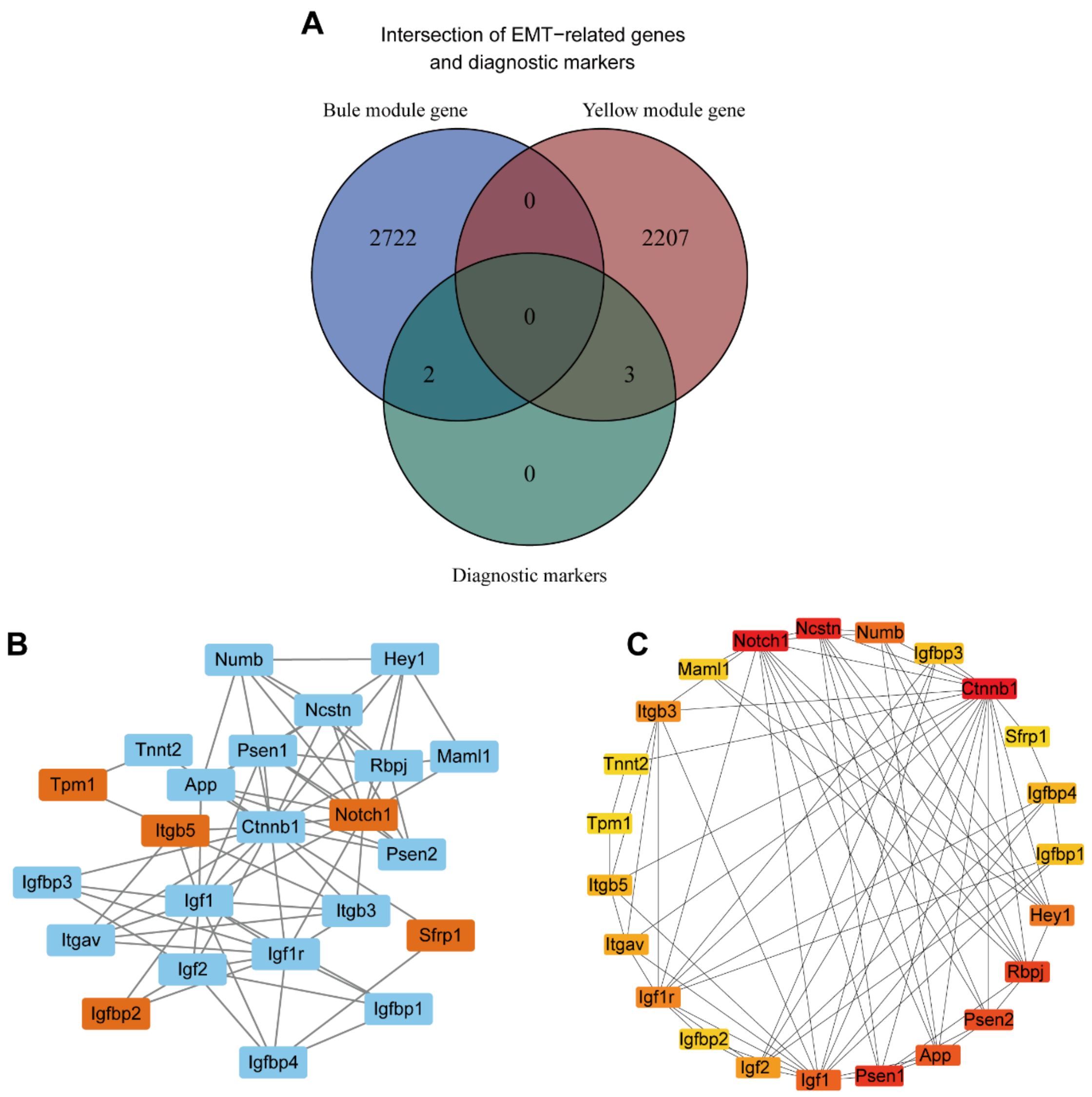

Supplementary Figure 2. Identification of hub genes. (**A**) Intersection of diagnostic markers with core module genes. (**B**) PPI network diagram of hub genes. (**C**) Network connectivity of hub genes. Darker red indicates that a gene is more connected in the network, while darker yellow indicates that a gene is less connected in the network.

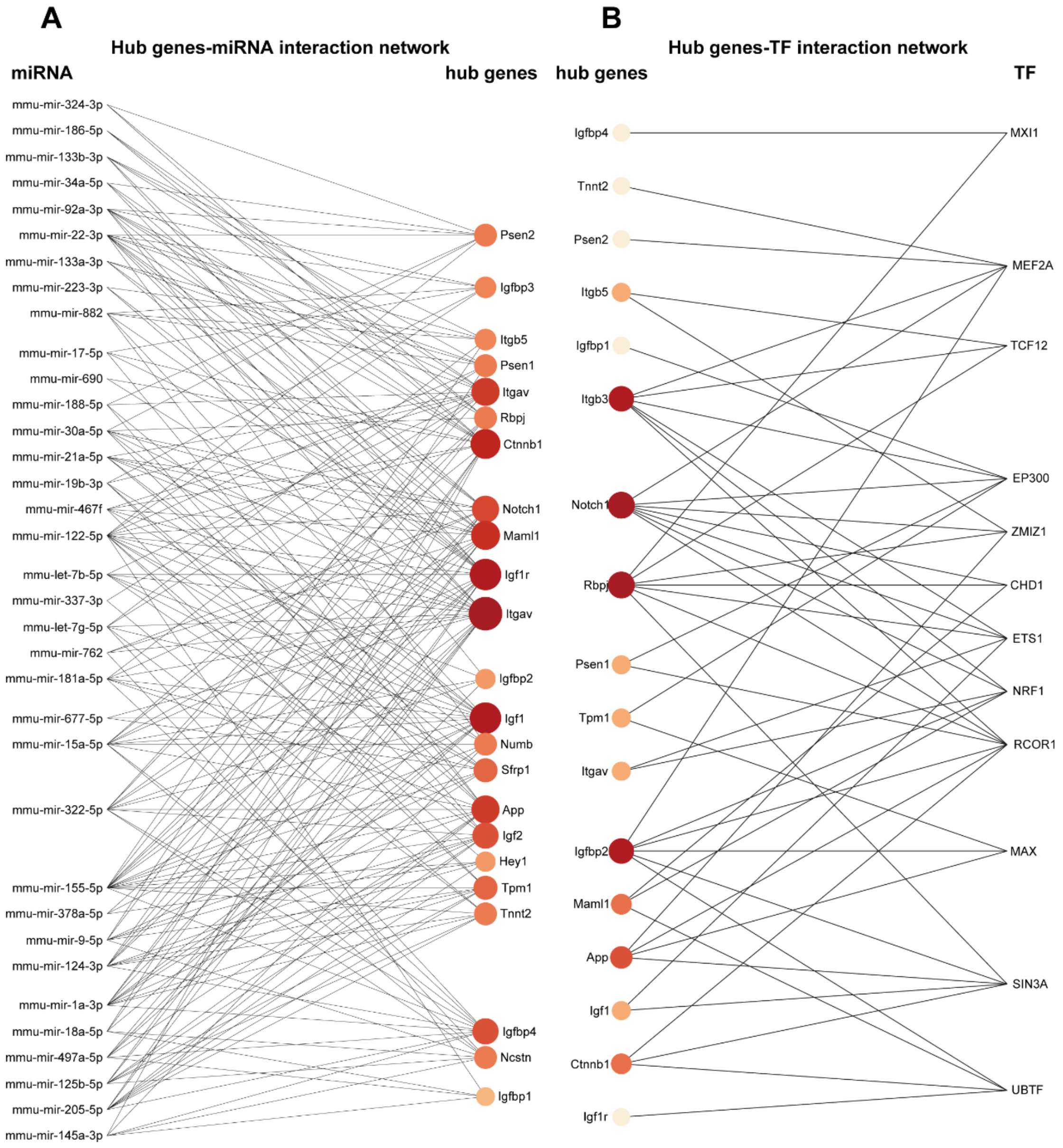

Supplementary Figure 3. Construction of interaction networks with miRNAs or TFs based on hub genes. (**A**) Interaction network between hub genes and miRNAs. (**B**) Interaction network between hub genes and TFs. The color intensity corresponds to the hub gene connectivity.

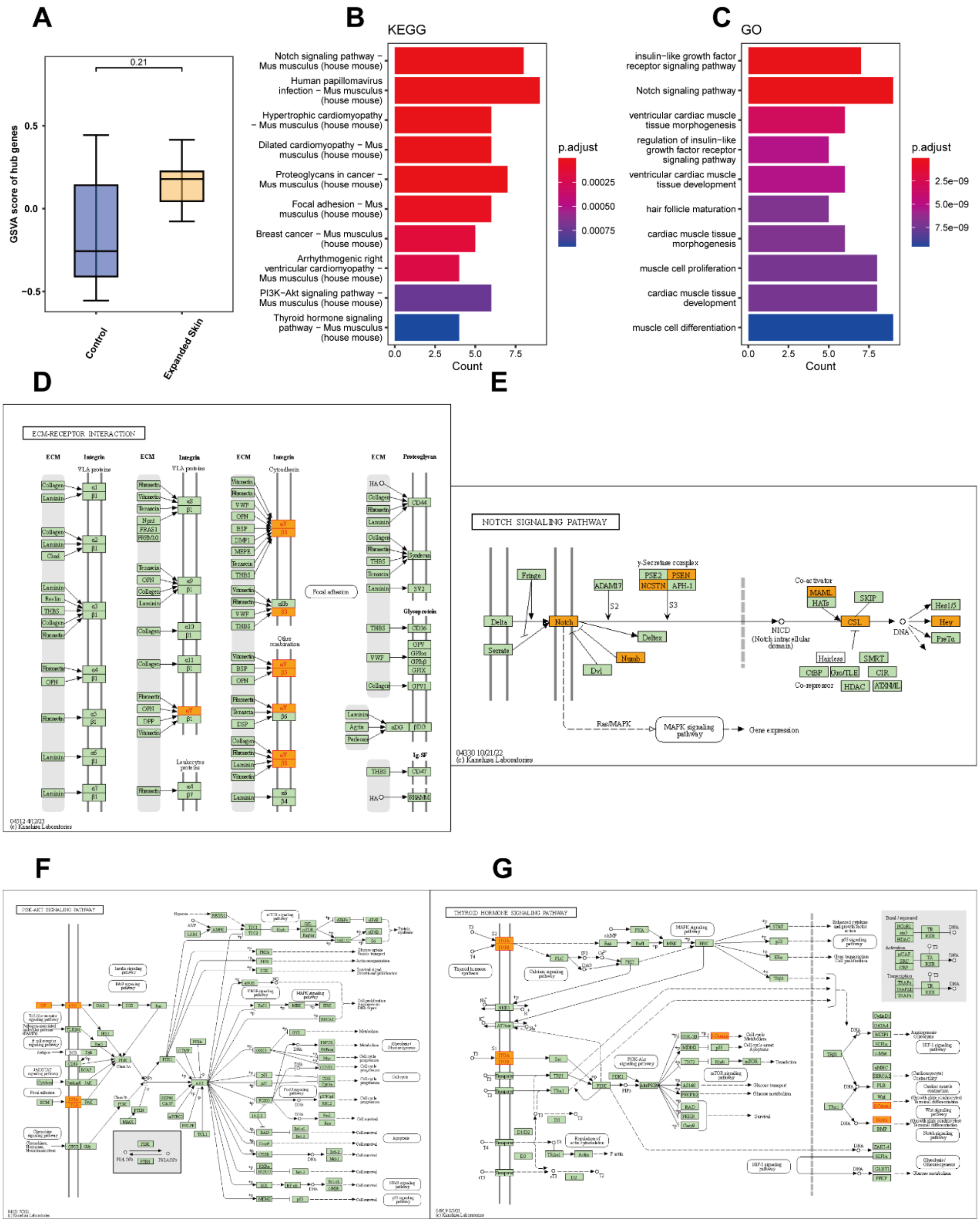

Supplementary Figure 4. Hub gene enrichment analysis based on the integrated dataset. (A) Differences in GSVA scores of hub gene sets between two groups. (**B**) KEGG enrichment results of hub genes. (**C**) GO enrichment results of hub genes. (**D**) KEGG enrichment pathway map of ECM-receptor interaction. Orange boxes indicate hub genes enriched in this pathway. (**E**) KEGG-enriched pathway map of Notch signaling pathway. Orange boxes indicate hub genes enriched in this pathway. (**F**) KEGG

enrichment pathway map of the PI3K-AKT signaling pathway. Orange boxes indicate hub genes enriched in this pathway. (**G**) Thyroid hormone signaling pathway. Orange boxes indicate hub genes enriched in this pathway. GSVA, Gene set variation analysis; KEGG, Kyoto Encyclopedia of Genes and Genomes; GO, Gene Ontology.

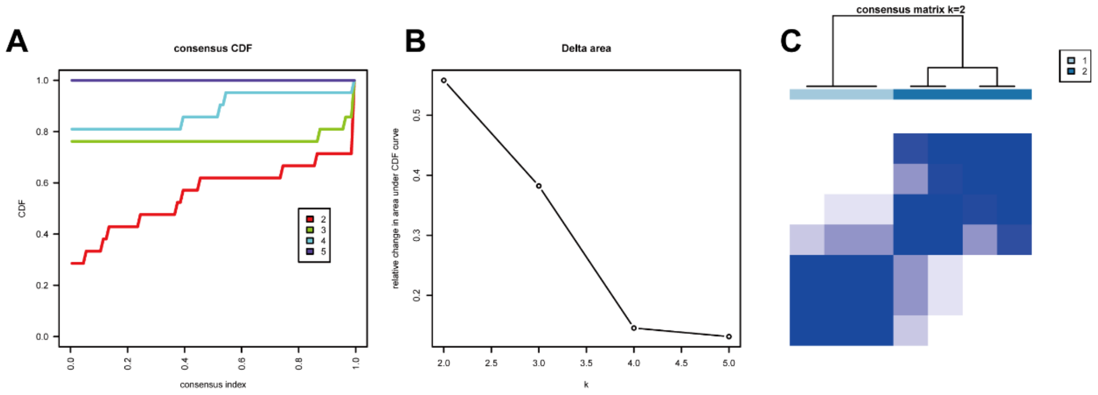

Supplementary Figure 5. Identifies skin growth subtypes based on the integrated dataset. A. Area under the cumulative distribution curve for different values of k. B. Cumulative distribution curve for different values of k. C. Sample clustering heat map for k=2.

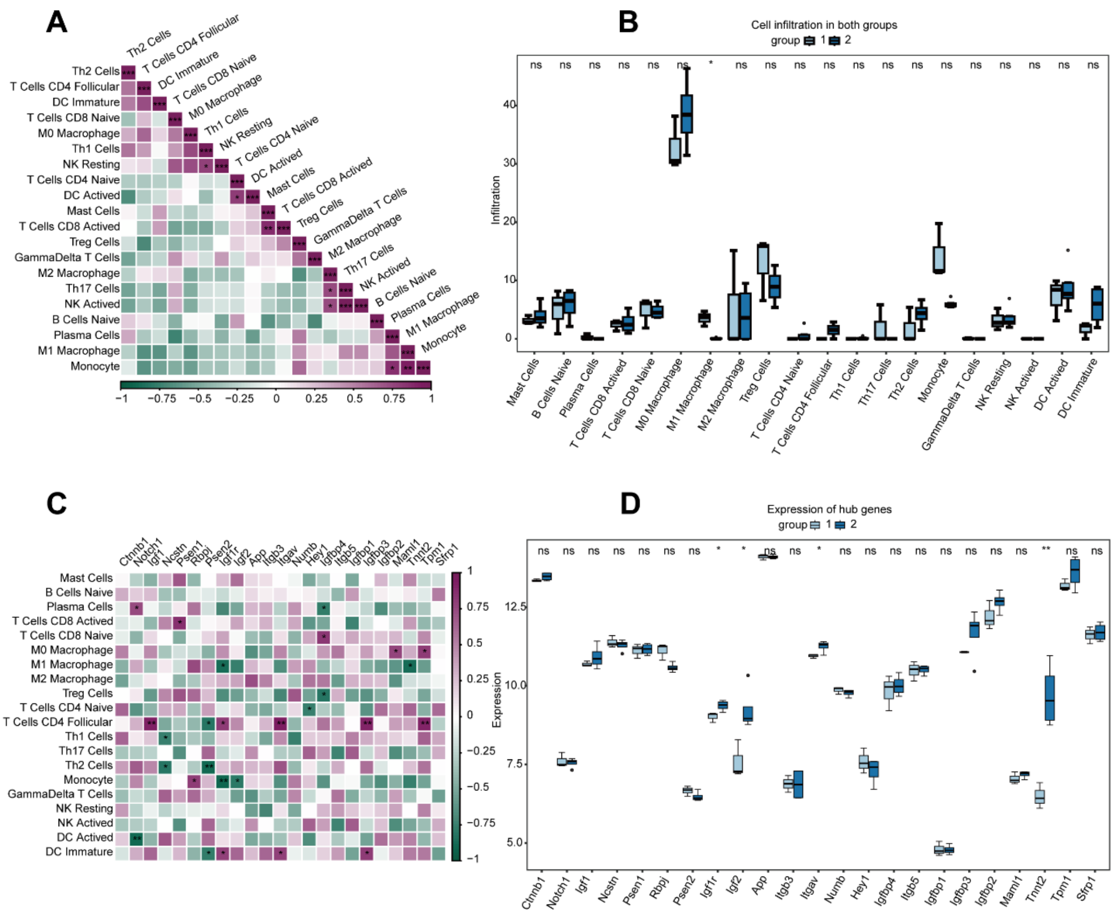

Supplementary Figure 6. Analysis of skin growth subtype immune infiltration based on the integrated dataset. A. Correlation between 25 kinds of immune cell infiltration. Purple indicates positive correlation and green indicates negative correlation. B. Differences in infiltration of 25 immune cells between the two subtypes. C. Correlation between the expression of hub genes and the infiltration of 25 immune cells. Purple indicates positive correlation and green indicates negative correlation. D. Differences in the expression of hub genes between the two subtypes of skin growth. Asterisks indicate significance levels: * p< 0.05, ** p< 0.01, *** p< 0.001, ns indicates not significant.

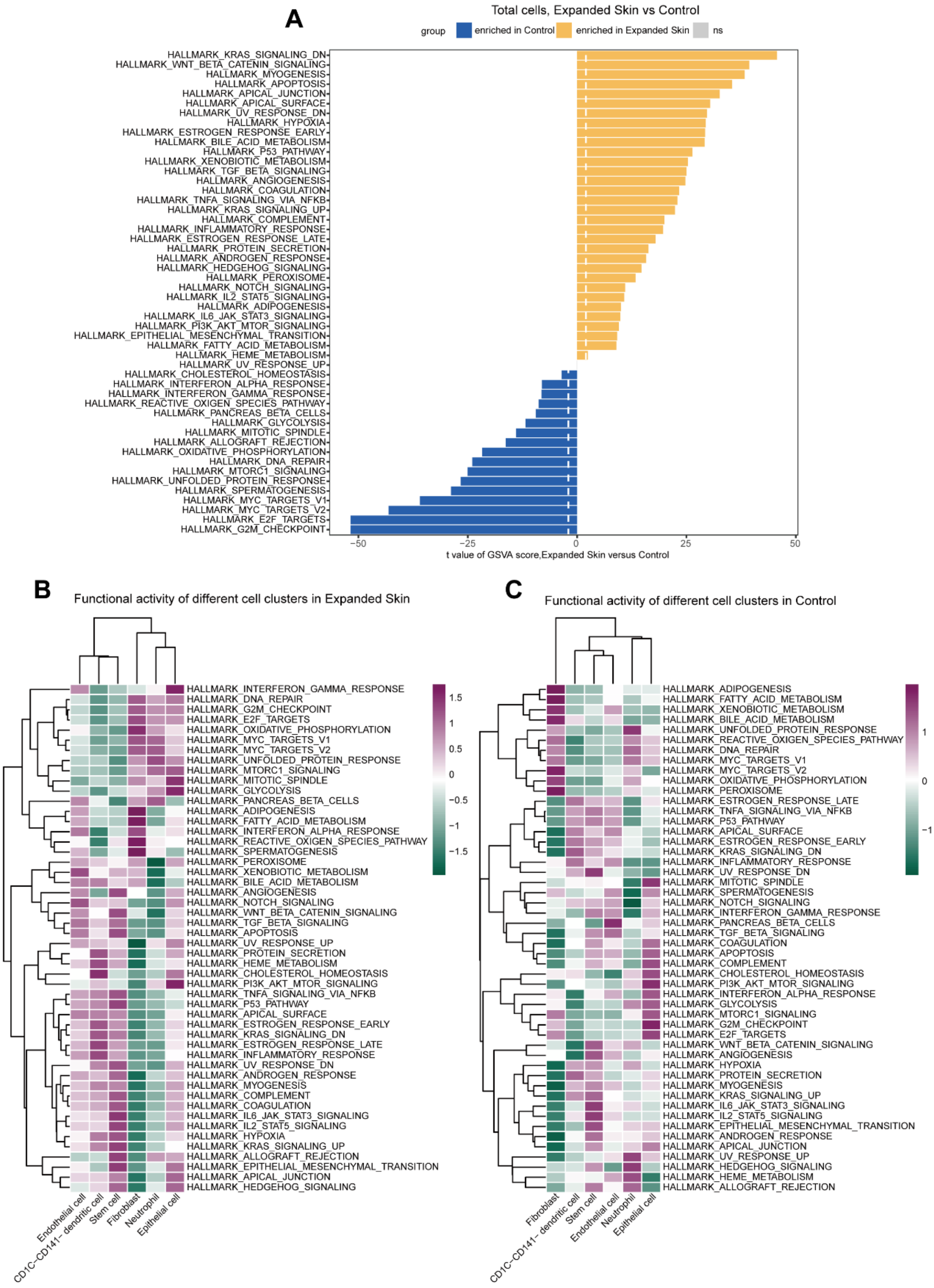

Supplementary Figure 7. Analysis of the functional differences of different cell clusters based on the GSE146637 dataset. (**A**) Functional differences in all cells in expanded skin and control groups. Blue bars: gene set enrichment activity is significantly upregulated; yellow bars: gene set enrichment activity is significantly upregulated; gray bars: gene set enrichment activity is not significantly different

between groups. (**B**) Functional differences in different cell clusters in expanded group. (**C**) Functional differences in different cell clusters in control group. Purple: upregulation of functional activity; green: downregulation of functional activity.

References

1. Wilkerson MD, Hayes DN. ConsensusClusterPlus: a class discovery tool with confidence assessments and item tracking. *Bioinformatics*. 2010;26(12):1572-1573.
